# A Supramolecular Ferroelectric With Two Sublattices and Polarization Dependent Conductivity

**DOI:** 10.1002/advs.202510258

**Published:** 2026-02-11

**Authors:** H. Mager, M. Litterst, Sophia Klubertz, Shyamkumar V. Haridas, Oleksandr Shyshov, M. von Delius, M. Kemerink

**Affiliations:** ^1^ Institute For Molecular Systems Engineering and Advanced Materials Heidelberg University Heidelberg Germany; ^2^ Institute of Organic Chemistry University of Ulm Ulm Germany

**Keywords:** ferroelectricity, electrical conductivity, supramolecular polymers, small molecules

## Abstract

The possibility to combine and finetune properties of functional molecular materials by chemical design is particularly relevant for organic ferroelectrics. In this work, we investigate a class of organic molecular materials that show long‐range supramolecular organization into fibrillar bundles. In solid state, the material shows ferroelectric behavior resulting from two largely independent dipolar moieties that show up as two separate coercive fields in polarization‐hysteresis and capacitance‐voltage curves. Moreover, the material shows a long‐range electronic conductivity that arises due to oxidation at the positive electrode, followed by electron transfer between neighboring molecules. We find that this conductivity is modulated by the direction and degree of ferroelectric polarization, which we interpret in terms of injection barrier modulation at low electric fields and a recently developed framework for asymmetric polaron hopping at high fields. With two distinct, partially independent dipolar moieties offering the possibility to use ferroelectric properties to modulate conductance, the materials presented herein are a promising basis for multifunctional materials.

## Graphical abstract

Ferroelectric behavior in a supramolecular material with two, independently switchable, sublattices is demonstrated. The sublattices can be associated with the two dipolar groups in the compound. Moreover, the polarization is found to couple to the charge transport in the material, giving rise to a switchable, directional conductivity.

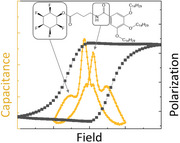

## 1 Introduction

Ferroelectricity was first discovered in Rochelle salt by J. Vasalek in 1921 [1], opening up an extensive field of research. Ferroelectric materials are characterized by a bistable electric polarization that is switchable by application of an external electrical field. Furthermore, all ferroelectric materials are also piezo‐ and pyroelectric, making them relevant for many applications, including microphones, transducers, actuators, sensors, and memory chips [2, 3, 4]. While Rochelle salt contains ions of the organic tartaric acid, the following years of research and discoveries were dominated by purely inorganic materials. Barium titanate (BTO) and lead zirconium titanate (PZT) emerged as two of the leading candidates for applications, owing to their high polarization values, fast switching, high retention, and stability [5, 6, 7, 8, 9]. For organic materials, the co‐polymer poly(vinylidene fluoride‐trifluoroethylene) P(VDF‐TrFE), consisting of vinylidene fluoride (VDF) and trifluoroethylene (TrFE), offers the best performance metrics and has even seen commercial use [10, 11, 12, 13, 14, 15, 16]. In general, organic ferroelectrics are flexible, solution processable, potentially energy‐efficient to produce, and lack toxic or rare elements. In the field of wearable electronics and for medical and biological applications, organic ferroelectric materials therefore enable unprecedented functionality, courtesy of their softness and non‐toxicity [17, 18, 19, 20]. However, organic ferroelectrics still lack in performance and stability compared to their inorganic counterparts.

While extensive research is done to find new organic ferroelectric materials with optimized parameters for existing applications, another direction is the (re)search for multifunctional materials. These materials combine a variety of optical, conductive, magnetic, ferroelectric, and other properties in one material [21, 22, 23, 24]. They are, on the one hand, of considerable interest with regard to the fundamental questions regarding the origin and the possible interplay of aforementioned properties. On the other hand, they may enable entirely new applications.

In this article we present a class of supramolecular ferroelectrics that show remarkable conductive properties, which depend on the polarization state of the material. A discussion of the origin and nature of the conductivity, which does not rely on the presence of a π‐system, has been reported in previous work [25]. In short, the dipolar polarization in the materials leads to a lowering of the energetic barrier at the (metal) electrode‐organic interface, enabling oxidation by electron extraction at the positive electrode and consecutive electron hopping between the molecules. The supramolecular polymerization of the material occurring at elevated fields and temperatures enhances the conductivity significantly. Here, we focus on the experimental ferroelectric characterization, including experimental evidence for the existence of two different ferroelectric sub‐lattices, and the interplay of ferroelectric polarization and conductivity. As it turns out, the coupling between both properties presents a challenge when trying to extract common ferroelectric parameters. We present different approaches to overcoming these hurdles and how to obtain approximate values for relevant ferroelectric parameters like the coercive field. By comparing ferroelectric behavior of molecular derivatives, we assign individual contributions, appearing as separate coercive fields, in ferroelectric measurements to the corresponding ferroic sublattice.

Conductive switching in organic ferroelectrics has been shown in semiconducting materials, that is, materials that contain an extended π‐system, for the injection‐limited [26] and the bulk‐limited cases [27]. While the latter determines a property of the active material only, the former determines a device property that is determined by the combination of active and electrode materials in the device. Casellas et al. even demonstrated the coexistence of both mechanisms in a single device [21]. Although similar in phenomenology and potential applications, these processes are fundamentally different from those governing ferroelectric tunnel junctions, which have the drawback of requiring extraordinary thin films [28]. In all of these examples, the ferroelectric polarization modulates the conducting properties of the charge‐carrying material, either by changing the energetic barriers at the (metal)electrode‐organic interface or directly influencing the bulk conductivity by introducing an asymmetric potential into site energies involved in charge hopping [27, 29]. In principle, this multifunctionality allows the creation of memory cells based on crossbar arrays sandwiching just one (organic) material, simplifying the architecture of memory devices [16].

## 2 Results and Discussion

**FIGURE 1:**
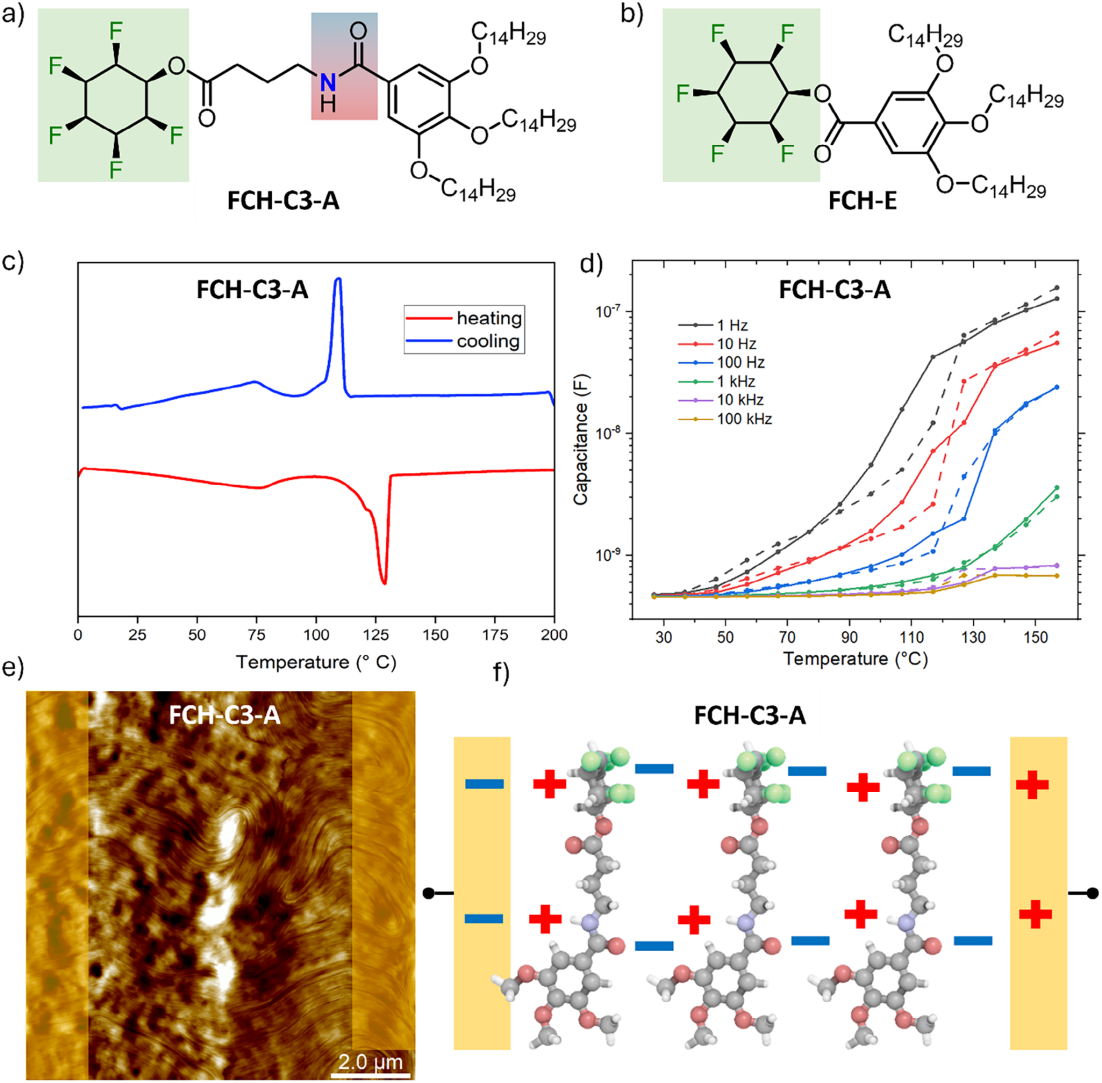
Molecular structures of (a) **FCH‐C3‐A** and (b) **FCH‐E**. (c) Differential scanning calorimetry traces of **FCH‐C3‐A** measured under a nitrogen atmosphere. (d) Dielectric spectroscopy measurements at various frequencies, where solid lines signal heating and dotted lines cooling traces. (e) AFM topography of annealed **FCH‐C3‐A** with the location of the buried metal electrodes indicated. Full color scale: 70 nm. (f) Schematic illustration of supramolecular stacking of the building blocks within a **FCH‐C3‐A** fiber between electrodes, showing the plausible orientation of the two dipolar moieties. Red, blue and green indicate oxygen, nitrogen, and fluorine atoms, respectively. Molecular structure optimized using the Universal Force Field (UFF).

The investigated molecules are shown in Figure 1a,b, and are abbreviated as **FCH‐C3‐A** and **FCH‐E**, respectively. Full chemical names and information on the synthesis procedure are given in Supporting Information S1: Section 1. **FCH‐C3‐A** consists of a phenyl ring attached to long alkyl chains that facilitate solubility. More importantly for this work, the molecule contains two strong dipolar units, an all‐*cis* 1,2,3,4,5,6‐hexafluorocyclohexane unit (FCH, 6.2 Debye) and an amide group (A, 3.7 Debye) that is connected through a three‐carbon (C3) spacer. The predicted dipole moments are for single monomers, while for head‐to‐tail stacked units, the net dipole moment will depend on the number of units stacked and is expected to exceed the sum of its units [30, 31]. The **FCH‐E** reference molecule lacks the amide‐C3 bridging unit of **FCH‐C3‐A**, but is otherwise structurally identical. Shyshov et al. showed previously that **FCH‐C3‐A** can undergo non‐covalent polymerization in solution, leading to the formation of supramolecular double helices that are transferable to the solid state by simple spin coating on a substrate [32]. We note that due to the presence of (‐CHF‐) groups, all‐*cis* fluorinated cyclohexanes do not belong to the group of compounds referred to as “PFAS” and that several biomedical studies have not given rise to any concerns about unspecific toxicity [33, 34, 35, 36].

We observed similar behavior in solid thin films, where field annealing at elevated temperatures results in the formation of supramolecular fibers aligned in the direction of the applied external field, as shown by the AFM height image in Figure 1e. This is reminiscent of the formation of supramolecular columns in the ferroelectric liquid‐crystalline benzene‐1,3,5‐tricarboxamide (BTA), where the long‐range order facilitates long‐range dipolar alignment [37, 38]. For **FCH‐C3‐A**, a schematic depiction of the possible orientation of the dipolar moieties in a supramolecular fiber is shown in Figure 1f. Hence, after fiber formation, the two dipolar moieties are assumed to form spatially separated sub‐stacks [32]. The supramolecular polymerization in **FCH‐C3‐A** has been shown in previous work to enhance the conductivity of the material significantly [25]. Its influence on the ferroelectric properties is investigated below.

As **FCH‐C3‐A** and **FCH‐E** tend to form rough and not completely closed layers, and to facilitate topographical and structural investigations, patterned interdigitated electrodes (IDEs) with micrometer‐sized gaps were used. Thin films were created by drop‐casting and measured in ambient conditions unless noted otherwise. Although not explicitly studied here, we found that ferroelectric and conductivity measurement results were very similar in ambient or nitrogen atmosphere or in vacuum. In‐plane electrodes and rough films make an exact quantitative measurement of current density and, therefore polarization difficult. Hence only measured currents are shown, and further dependent parameters are estimated. Likewise, the given electric field values (voltage/gap width) are approximate, as the in‐plane geometry leads to bending of the field lines, which results in deviations in field strength compared to more symmetric out‐of‐plane parallel plate capacitor geometries. A detailed summary of sample fabrication, layout, and characterization is given in Supporting Information S1: Section 2.

Ferroelectric materials commonly exhibit a phase transition from a higher temperature paraelectric phase to a lower temperature ferroelectric phase at the Curie‐temperature, at which a structural transition from a state of higher symmetry to a state of lower symmetry occurs. The ferroelectric‐to‐paraelectric phase transition can be detected experimentally by various means, such as differential scanning calorimetry (DSC) [39], dielectric spectroscopy (DS) [37, 39], second‐harmonic generation (SHG) [40] and polarization switching measurements [41]. DSC measurements for **FCH‐C3‐A** are shown in Figure 1c. Two endothermic peaks are observed in the heating trace, a small but broad one centered around 70°C and a larger one at around 127°C. The latter one is readily identified as the melting point of the material, while the smaller one implies another structural transition that will (below) be identified as associated with the mobilization of the amide group.

Ferroelectric materials commonly exhibit Curie‐Weiß‐like behavior of the electric permittivity, measurable by DS. Figure 1d shows the measured capacitance, which is a sufficient proxy for the permittivity, of an **FCH‐C3‐A** coated IDE, depending on temperature for various frequencies. In contrast to the DSC measurement, we do not find peak‐like features in the heating trace, instead a gradual increase of the capacitance with increasing temperature is observed, followed by a large jump to higher capacitances around the melting point. Since no Curie‐Weiß singularity is observed at that temperature, we conclude that the DSC peak at 70°C is of a non‐ferroic nature, consistent with ferroelectric behavior found above 70°C, as discussed in detail below. The absence of Curie‐Weiß‐like behavior does not exclude ferroelectricity in materials, but implies that if proven ferroelectric, the Curie temperature of both compounds investigated here lies at temperatures at or above the melting point. This has been observed before, for example, in P(VDF‐TrFE) or in the high‐temperature ferroelectric phase of BaMnF_4_ [42, 43]. We explain the gradually increasing capacitance by a gradual mobilization of dipolar moieties with rising temperature, and therefore available thermal energy, in the system, which is in line with the fact that the capacitance upswing coincides with the DSC peak at 70°C. Another, not mutually exclusive possibility is the material approaching the transition to the paraelectric state but melting before reaching the transition temperature. At the melting point, a large enhancement in dipolar mobility leads to a steep increase in capacitance. Lower modulation frequencies (1–100 Hz) give rise to higher amplitudes already at lower temperatures, which implies relatively slow‐moving dipoles that are difficult to excite at higher frequencies.

In the following, we present polarization switching measurements on films of **FCH‐C3‐A** and **FCH‐E** using the double‐wave‐method (DWM) [41] that in principle allows extracting of the pure switching current by correcting for background currents caused by leakage and displacement. Subsequent integration then yields the ferroelectric polarization versus field loops.

**FIGURE 2:**
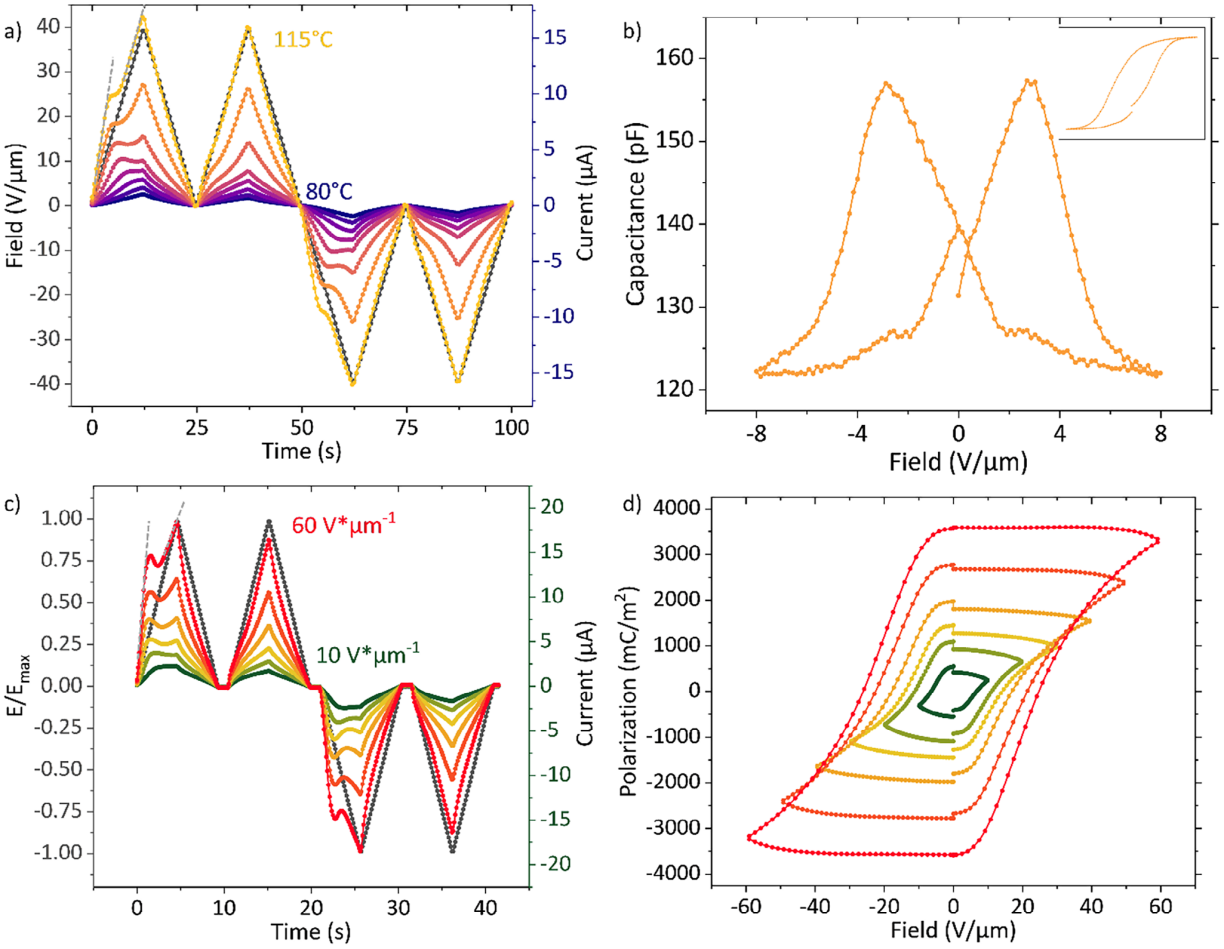
(a) Double wave measurements at constant field (40 V µm^−1^) and frequency (10 mHz) of **FCH‐C3‐A** for varying temperatures. The dashed grey lines are linear approximations of the slopes below and above the coercive field. (b) Shows a low field CV measurement at 110°C with the reversible part of the polarization obtained from integration shown in the inset; the vertical scale amounts to ± 0.23 mC m^−2^. (c) Double wave measurements at constant temperature (100°C) and frequency (25 mHz) for varying applied fields in. (d) “Polarization” hysteresis curves obtained by integrating the currents from c). The magnitude of the polarization and its non‐saturating behavior indicate they are not true ferroelectric polarization loops as discussed in the text.

In Figure 2a the response current to a DWM pulse at fixed amplitude and frequency is plotted for increasing temperatures. At around 90°C (shown in more detail in Figure S1), a shoulder starts to appear in the first and the third readout pulse. With increasing temperature, the shoulder evolves into a pronounced peak, that progressively shifts its center toward lower fields. The most straightforward origin of such a peak is ferroelectric polarization switching. Alternative explanations in terms of accumulation of mobile ionic species at the electrodes or material degradation would not cause a peak but rather continue with increasing bias. Moreover, the presence of any significant number of mobile ions, as well as of degradation, has been excluded in previous work [25].

The coercive field, which is the applied electric field where the bulk polarization completely inverts, is, in lowest order, identified as the field where the switching current is at its maximum. Ferroic switching in disordered organic molecules can typically be described as a thermally activated, nucleation limited switching (TA‐NLS) process [44, 45, 46]. The dependence of the coercive field on temperature and sweep frequency for **FCH‐C3‐A,** shown in Figures S2 and S3, shows qualitative agreement with the TA‐NLS theory as well as with experimental data for the well‐understood ferroelectric BTA [38]. The effect of the supramolecular fiber formation is seen in Figure S4, where repeated DWM measurements lead to a continuously increasing conductivity, as well as a shift of the coercive field to higher fields. The latter can be explained in terms of the TA‐NLS model by an increase in critical nucleation volume, associated with a reduced structural disorder.

DWM measurements for varying maximum applied external fields are shown in Figure 2c. With increasing field strength, the switching peak takes form, decreases in width and its center is reached earlier in time. The peak center's shift comes to a stop at 30 V µm^−1^, a further increase of field strength solely results in a slight narrowing of the peak and a strong increase in background current relatively to the peak amplitude. This peak evolution with field strength is typical for ferroic materials, as for fields below the coercive field, only a subset of dipoles is flipped, according to the Preisach distribution of the material [47]. Once the coercive field is reached, all existing dipoles are flipped and the peak growth saturates, resulting in the saturation of the polarization when applying progressively increasing electric fields. However, the hysteresis loops associated with the currents from Figure 2c show an ever‐growing “polarization” with field, as seen in Figure 2d. Furthermore, the polarization values are multiple orders of magnitude above the theoretically estimated polarization of approximately 83 mC m^−2^. This raises the question whether the material is truly ferroic, as leaky dielectrics can also show ferroelectric‐like polarization loops [48]. However, the occurrence of current peaks at well‐defined fields below the maximum applied field, and these peaks being describable in terms of the TA‐NLS model, conflicts with the leaky‐dielectric hypothesis.

A further sign of truly ferroic behavior can be found in capacitance–voltage (CV) measurements, where a DC‐bias with a superimposed AC‐modulation is swept back and forth, where the latter is used to measure the (small signal) capacitance. Ferroelectric materials possess a heightened dipolar sensitivity to external perturbations in vicinity of the coercive field, where the permittivity increases drastically. This results in a characteristic butterfly loop of the capacitance, with peaks in increasing field direction for both polarities. The coercive fields obtained from CV loops generally differ from the ones obtained from DWM measurement due to the different sweep rate for both experiments. A CV measurement of **FCH‐C3‐A** is shown in Figure 2b and demonstrates the butterfly shape that is characterisitc for ferroelectrics. Integrating the CV‐loop yields a well‐shaped hysteresis loop, reflecting the reversible polarization that is generally only a minor fraction of the total polarization [11]. Hence, we conclude that **FCH‐C3‐A** is a true ferroelectric. The presence of macroscopic dipoles in this material is further corroborated by piezoresponse force microscopy measurements in the SI. Therefore, the overestimation of the polarization has to result from the conducting properties of the **FCH‐C3‐A**, which apparently cannot be accounted for with the simple DWM correction that relies on the assumption that any background current does not depend on sample history and, as such, not on polarization. As discussed in a later section, the observed behavior is consistent with a polarization‐modulated conductivity. Additional attempts of correcting for the conductivity as well as the corresponding hysteresis loops to the current measurements are presented in Figure S5.

**FIGURE 3:**
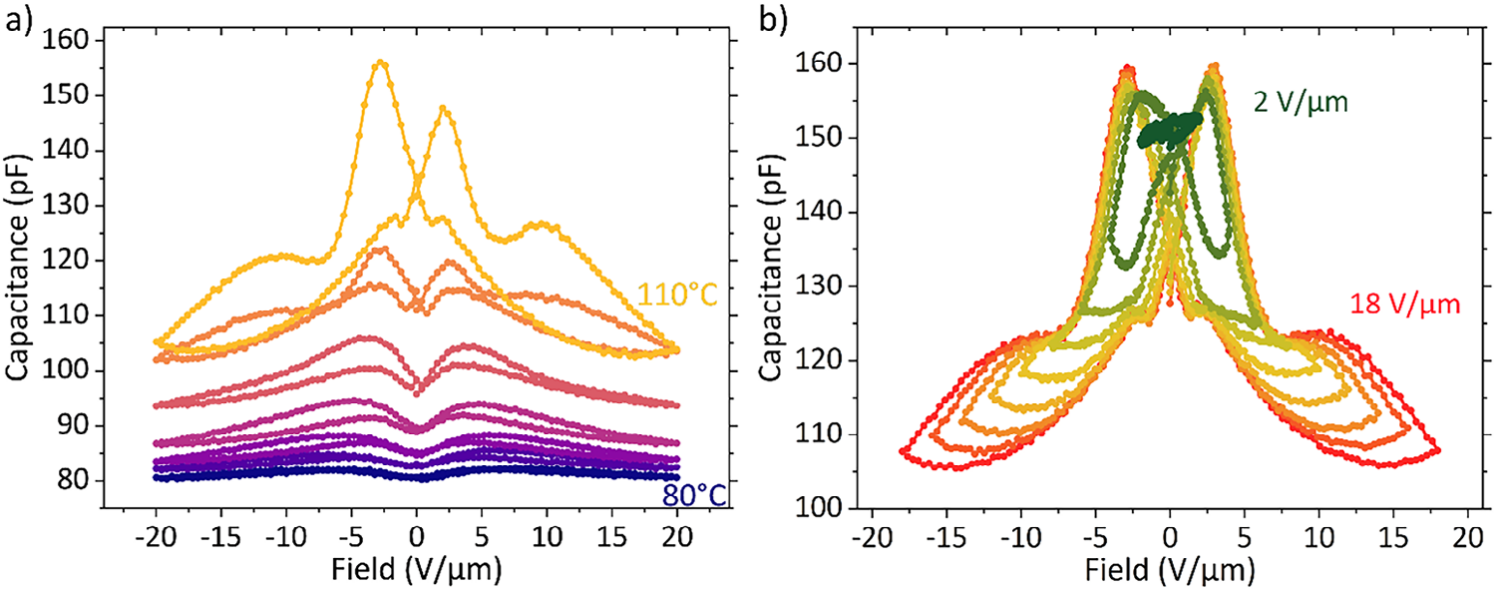
Capacitance‐voltage measurements of **FCH‐C3‐A**, measured (a) at fixed maximum DC‐field (20 V µm^−1^) for increasing temperatures (Δ*T* = 5°C) and (b) at fixed temperature (110°C) for increasing maximum DC‐field strength (Δ*V* = 2 V µm^−1^). Both measurements are taken with a small signal frequency and amplitude of 100 Hz and 0.2 V µm^−1^, respectively.

Additional insight into the switching process of **FCH‐C3‐A** could be obtained by sweeping a larger parameter space with CV measurements, see Figure 3a. Around 85°C, the butterfly shape begins to emerge with its peaks becoming more pronounced and shifting to lower fields with rising temperature, in a similar fashion as in the DWM measurements. Interestingly, at 105°C a second feature appears on the flanks of either side of the butterfly loop, becoming more pronounced at 110°C. As a natural explanation for the occurrence of a second peak, the presence of two dipolar moieties, the all‐*cis* fluorinated cyclohexane ring (FCH) and the amide group (A), in the **FCH‐C3‐A** comes to mind. This would require the spatially separated stacks of these groups to act as two, at least partially independent ferroelectric sublattices with separate coercive fields. To our best knowledge, there is no existing literature regarding organic materials with two separate ferroelectric lattices, and there are no CV loops reported for such systems. Our CV loops are reminiscent of CV measurements on lead zirconium titanite (PZT) conducted by Bar‐Chaim et al. [49]. who attributed double peak butterfly loops to the presence of 90° and 180° domains, which possess different coercive fields.

The field dependence is investigated further in Figure 3b, where CV curves for a stepwise increasing maximum applied DC‐bias are depicted. The separate contributions of the two sublattices can be discerned. For small fields, incomplete loops are observed, as neither of the coercive fields is reached. From ∼8 V µm^−1^ on, a well‐formed complete butterfly loop is observed, and no further shift of the first peak is observed. The field of the peak maximum is the first coercive field with a magnitude of ∼2.8 V µm^−1^. At 10 V µm^−1^ the tails of the loops begin to broaden, eventually evolving into a complete second peak at ∼14 V µm^−1^ and beyond. The corresponding coercive field to the maximum of the second peak is around 9.8 V µm^−1^. Interestingly, once the second peak starts to develop, another small peak is observed around 1.9 V µm^−1^ in the direction of decreasing fields. A possible explanation is a field‐dependent Curie temperature (of the second sublattice), in line with what has been observed previously for BTO and P(VDF‐TrFE) and follows from the Landau‐Devonshire theory of ferroelectrics [50, 51]. For small fields and higher temperatures, these materials transition into a paraelectric phase, losing their polarization, which induces a peak in the CV curve for decreasing fields. However, as no corresponding features have been observed in the DWM measurements, we refrain from making a definite attribution.

Frequency‐dependent DWM measurements at 110°C displayed in Figure S3 similarly show two separate peaks that can be simultaneously described by the TA‐NLS model. The corresponding low‐frequency coercive fields of 3 to 5 V µm^−1^ for the low‐field peak and 10.5 to 12.5 V µm^−1^ for the high‐field peak agree well with the coercive fields observed in the CV measurements. This finding reinforces the notion that both the peaks are of ferroic nature and can be attributed to the switching of the two dipolar moieties. An assignment of the individual peaks to a specific moiety is discussed further below. Additionally, in low‐frequency, low‐field DWM measured at 110°C shown in Figure S6, it is possible to observe a transition from the first coercive field to the second, with a value of 1.7 V µm^−1^ for the former. Observing switching currents at two different fields qualitatively agrees with the findings from the CV loops, although it is difficult to discern at which fields exactly the transition from the switching of the first moiety to the switching of the second moiety occurs, which we attribute to a significant, disorder‐induced overlap of the switching peaks, in combination with the background currents owed to the conducting property of the material.

The molecule **FCH‐E** (see Figure 2b) is structurally very similar to **FCH‐C3‐A**, but is missing the amide group as well as the C3 spacer unit and, as such, might provide additional insight into the switching mechanism. In our previous work, **FCH‐E** was shown to be an insulator, only showing small leakage currents, with field annealing not resulting in the formation of long‐range‐oriented supramolecular structures; 2D GIWAXS and high‐resolution AFM measurements presented in Figure S7 do show the presence of pronounced structural order, albeit in the form of shorter supramolecular structures that cannot be long‐range oriented by an external field [25]. Moreover, as seen earlier in Figure S4, a fully developed long‐range supramolecular structure is no prerequisite for the ferroelectric properties of **FCH‐C3‐A**. In DSC and DS measurements on **FCH‐E**, only melting and freezing points are observed (see Figure S8). This suggests that the peak at 70°C in the DSC measurement of **FCH‐C3‐A**, and concomitantly the low‐field and low‐temperature response of the latter material, are associated with the (mobilization of the) amide group.

**FIGURE 4:**
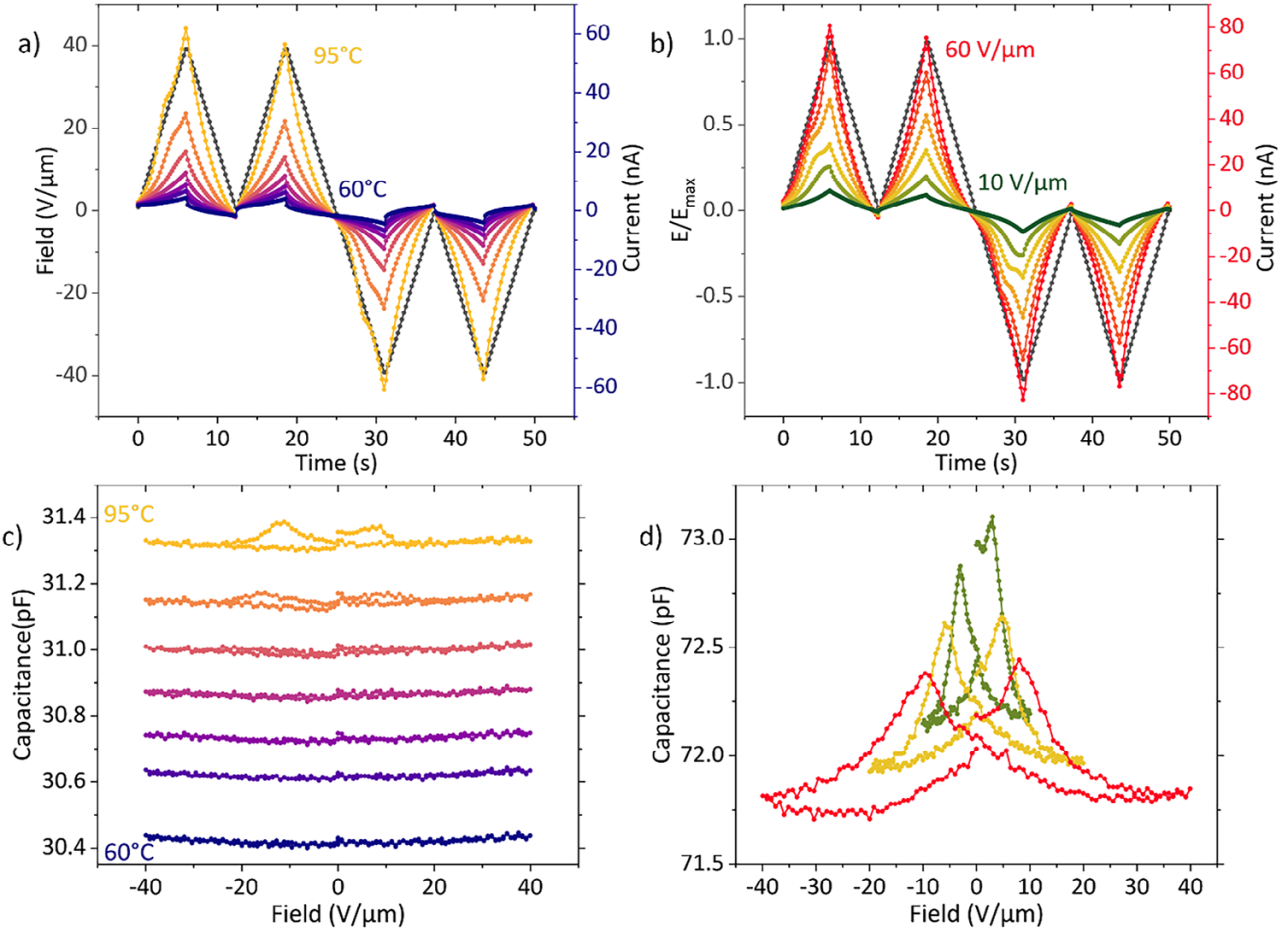
DWM measurements of **FCH‐E** at fixed frequency and maximum field for increasing temperatures (Δ*T* = 5°C) in (a) and at fixed frequency and constant temperature of 95°C for increasing maximum fields (Δ*E* = 10 V µm^−1^) in (b). The corresponding polarization hysteresis loops are shown in Figure S9. (c) CV loops taken with a small signal peak to peak amplitude of 1 V µm^−1^ and small signal frequency of 1 kHz for increasing temperatures (Δ*T* = 5°C). d) CV loops taken with a small signal peak to peak amplitude of 1 V µm^−1^ and a small signal frequency of 10 Hz for a stepwise increasing maximum field.

Figure 4a,b shows temperature‐ and field‐dependent DWM measurements of **FCH‐E** for fixed sweep frequency. The resulting polarization loops are presented in Figure S9a,d. Contrary to **FCH‐C3‐A**, the polarization shows indications for saturation at high fields and was limited to physically meaningful values, indicating a negligible contribution due to conductivity. The saturation polarization obtained from DWM measurements corrected for ionic contributions is around 5.1  ± 0.8 mC m^−2^ (see Figure S10), which is significantly lower than an approximate theoretical upper limit of 69 mC m^−2^ for a fully flipped all‐*cis* fluorinated cyclohexane group. This discrepancy is much larger than the uncertainty caused by, for example, film roughness (pk‐pk amplitude below ∼100 nm, compare Figure 1e, relative to film thicknesses of several µm) and suggests that only partial alignment of the dipolar moiety occurs, likely caused by insufficient molecular mobility, causing off‐axis dipole orientation or preventing switching altogether. Similar to **FCH‐C3‐A**, the temperature and frequency dependency of the coercive field are well described by the TA‐NLS law, see Figures S9c,d and S11c,d in the SI. A comparison of the fitting parameters with those obtained for the high field peak of **FCH‐C3‐A** show good agreement, reinforcing the assignment of the high field peak to the ferroelectric switching of the all‐*cis* pentafluorocyclohexane group.

The temperature‐ and field‐dependent CV loops of **FCH‐E** are presented in Figure 4c,d, respectively. In the former, ferroelectric butterfly loops are observed from 90°C on. The CV loops together with the DWM measurements are a strong indication of true ferroelectric switching in **FCH‐E**. In the field‐dependent loops (Figure 4d), the butterfly peaks continuously shift to higher fields with increasing applied maximum field. This is a result of the maximum applied field closing in on, and finally exceeding the coercive field, at which point the peak shift comes to a stop. Even at high DC‐fields and low small‐signal frequencies, only one peak is observed, supporting the hypothesis that the two peaks in the CV measurements of **FCH‐C3‐A** are caused by its two distinct dipolar moieties. Generally, the coercive fields for the **FCH‐E** obtained from DWM and CV loops are around 20 and 10 V µm^−1^, respectively. These values are of similar magnitude to the second, larger coercive field observed for the **FCH‐C3‐A**. This is consistent with the ascription made above that the smaller coercive field belongs to the amide group and, consequently, the larger coercive field belongs to the pentafluorocyclohexane group. This is also consistent with the intuitively expected lower steric hindrance for the re‐orientation of the amide group.

**FIGURE 5:**
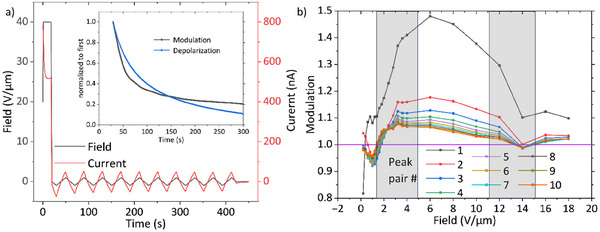
(a) Schematic of the zig‐zag conductivity switching measurements on **FCH‐C3‐A** films, where one rectangular poling pulse is followed by triangular pulses of alternating sign. These measurements correspond to one data point per peak pair in image (b), where the absolute peak ratio (conductivity modulation) for each peak pair is plotted against the triangular voltage amplitude. The overlaid gray bars indicate the approximate ranges of the coercive fields obtained from DWM and CV measurements. The inset to (a) plots the normalized current modulation together with the normalized depolarization current from the 40 V µm^−1^ poling pulse versus time.

In the final part of this article, evidence for polarization‐mediated switching of the charge injection and bulk conductivity in **FCH‐C3‐A** is discussed. To this end, we employed a triangular bipolar “zig‐zag” pulse sequence, where a single rectangular poling pulse is followed by multiple triangular pulses of alternating sign, therefore probing along and against the polarization direction. The conductivity modulation at the *n*th pair of negative and positive voltage peaks can then be calculated as the ratio of the corresponding absolute currents. A detailed showcase of the process including background current correction and error analysis is presented in Figure S12, and an alternative approach to eliminate the time delay between peaks via extrapolation in Figure S13. Any conductivity modulation is expected to vanish for triangular amplitudes above the coercive field. Likewise, linear response theory demands that no modulation occurs in the low‐field regime, as the derivative of the current‐voltage characteristic must be well‐defined at zero voltage. In addition, the presence of two different coercive fields has to be kept in mind. Figure 5a shows a schematic of the zig‐zag measurement, which was done for varying maximum triangular amplitudes.

To start with, the effects of depolarization, that is the relaxation of the dipolar ordering over time, have to be considered. Depolarization of the material is greatly accelerated at 100°C and results in a current of opposite sign compared to the poling pulse, which is included in the measured currents and hence is superimposed on the possible conductivity modulation. The associated currents were previously shown to be small in the used materials; the same holds for any spurious ionic contributions to the current transient [25]. The current transient due to depolarization and any ions can be extracted from the zero crossings of the zig‐zag measurements and is shown in Figure S12 for the 1.8 V µm^−1^ triangular maximum field. After correcting for these background currents, the ratio of each negative triangular pulse peak to its successive positive counterpart is taken and plotted in Figure 5b against the applied maximum triangular field, for all peak pairs. Over the change from the first to the last peak pair, the effect of the polarization decay is observed, resulting in a decrease in the polarization modulated conductivity. Since the depolarization current falls to 50% of its starting value in 10 s (see Figure S12c), the modulation concomitantly strongly decreases over time. In the inset of Figure 5a, the normalized depolarization current and the normalized modulation are plotted in parallel, showing a clear correlation between (de)polarization and conductivity modulation, suggesting a causal relation [52].

As mentioned above, the bulk conductivity modulation is a higher‐order effect and should vanish at low fields, that is, converge to a value of unity. Surprisingly, prior to turning positive, the modulation first becomes smaller than one. We attribute this to injection barrier modulation (IBM), where currents in the polarization direction are increased, which experimentally was observed at low fields [21, 26]. Irrespective of that, the successive increase in modulation to values over unity is indicative of bulk conductivity switching (BCS), where the conductivity is increased when field and polarization are antiparallel and vice versa [21, 52]. As such, IBM and BCS are competing effects, with opposite ‘polarity’ in terms of which current direction, relative to the polarization direction, is largest. Importantly, while field inhomogeneity due to, for example, fringing effects in our in‐plane devices may broaden the field dependence, this will not affect the polarity, which therefore may be used as an indicator for the nature of any conductivity modulation. It was shown that in materials exhibiting both effects, IBM is observed at low fields, while BCS begins to dominate at intermediate to high fields. In our previous work, we demonstrated the existence of a substantial injection barrier between the Au electrodes and the active material, leading to the necessity to (locally) align the molecular dipoles with the electric field at the injecting contact in order to obtain an appreciable, and potentially non‐limiting charge injection [25]. Hence, some form of IBM is expected at low field. Note that the coercive field is a bulk material property and that the reversal of individual dipoles in the likely more disordered contact regions is expected to occur already at substantially lower fields [53].

**FIGURE 6:**
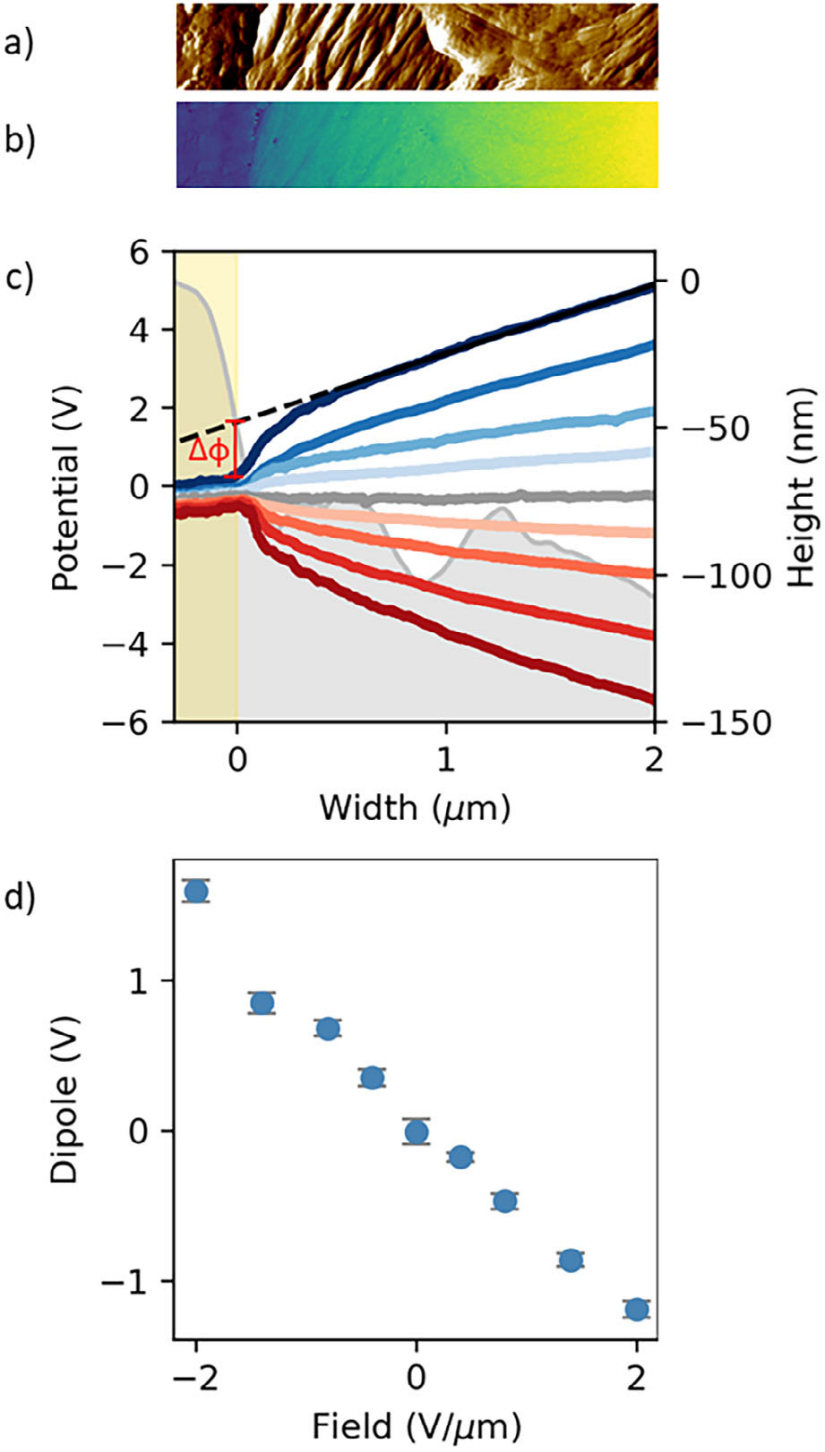
In‐operando KPFM measurements on a pristine **FCH‐C3‐A** device with 5 µm inter‐electrode gap. (a) Amplitude signal, showing the edge of the (biased) electrode on the left‐hand side; the (grounded counter electrode is not shown. (b) Corresponding surface potential measured in sideband mode. (c) Line sections of surface potentials at different applied voltages between +10 V (red) and −10 V (blue). The yellow shade indicates the electrode position. (d) Interfacial dipole versus field Δϕ, extracted as illustrated in (c).

To substantiate the assignment of the low‐field negative conductivity modulation to IBM, we performed in‐operando Kelvin probe force microscopy (KPFM) measurements of the (spatial distribution of the) electrostatic potential in the channel. The results are shown in Figure 6. For finite applied fields, we observe step‐like features at both contacts that are superimposed on a more or less linear background. At low fields, the voltage steps increase linearly with field and are of the same magnitude at both contacts, as expected for field‐induced interfacial dipoles. For the shown fields, their magnitude remains below the estimated injection barrier between Au and **FCH‐C3‐A** of ∼1.7 eV [25]; instrument limitations do not allow to measure beyond 2 V µm^−1^ (+/‐10 V), preventing the direct observation of the expected saturation of the interfacial dipole, either at the value needed to make the contact Ohmic, or at the value set by the available dipoles. The former and latter would be slightly below 1.7 eV and around 2 eV, respectively [25]. Importantly, the measured interfacial energy shifts are of a magnitude and occur in a voltage range that is fully consistent with our interpretation of the negative current modulation in Figure 5b in terms of injection barrier modulation.

In Figure 5b, beyond ∼2 V µm^−1^, depending on peak pair, the steady decrease in modulation with increasing field can be explained by bulk conductivity modulation with a continuously increasing fraction of reversed polarization. A further, significant drop to unity is observed at 14 V µm^−1^, which is in the range of the second coercive field. From here on, the polarization is switched back and forth every triangular pulse and the modulation vanishes. The peak ratio being slightly above unity beyond the coercive field can be explained by polarization imprint effects, where a portion of hysterons becomes permanently trapped in one state, which can result from the asymmetric poling procedure [54]. Importantly, the qualitative shape of Figure 5d, and particularly the (near) vanishing of the modulation beyond the highest coercive field, excludes more trivial explanations in terms of, for example, ionic currents.

Although **FCH‐C3‐A** is not a conventional organic semiconductor in that it lacks an extended π‐system and charge transport accordingly involves a more general mechanism of subsequent oxidation and reduction of the energetically most accessible state, the observed behavior fits well with observations and simulations of BCS in ‘conventional’ semiconducting organic ferroelectrics by Gorbunov et al. [27] and Casellas et al. [21]. The former modeled BCS as Marcus hopping between discrete states in an asymmetric potential landscape, modulated by the ferroelectric polarization. The mechanism was later expanded by Johann et al., combining molecular dynamic simulations and density functional theory [21, 29].

An alternative and potentially more direct way of estimating the conductivity modulation is by taking the ratio of the slopes of the current response versus increasing field in the first (anti‐parallel current and polarization) and second (parallel current and polarization) peaks of the DWM measurement, compare the dashed grey lines in Figure 2a,c. This modulation factor, by its definition, is also the modulation factor required to explain the abnormally high “polarization” in Figure 2d. The modulation data obtained from the current slopes as well as the corresponding fits are shown in Figure S14. Disregarding low fields and temperatures, where incomplete switching results in reduced slopes after the coercive field and therefore exaggerated modulation factors, the saturated on/off‐ratios are between 2 and 5, depending on the exact measurement parameters. These exceed the maximum modulations in Figure 5b and Figure S13d that are around 1.3 to 1.5. The discrepancy can be explained by the effects of depolarization affecting the zig‐zag measurement. The depolarization current, see Figure S12c, reduces by a factor ∼3 after 30 s, implying, in lowest order (cf. inset of Figure 5a), a reduction of the remaining polarization by a similar amount. Hence, the depolarization would reduce the on/off‐ratios obtained from the DWM slopes to ∼1.3–2.3, comparable to those of the zig‐zag measurements.

## 3 Summary and Outlook

In this work, we investigated the electronic properties of the multi‐functional small‐molecule material **FCH‐C3‐A**, which, in solid state, forms quasi‐1D supramolecular bundles. Combining CV and DWM measurements, we showed that thin films of this material have ferroelectric properties, brought about by two largely independent polar lattices that are formed by spatially separated sub‐stacks of the two dipolar groups in the **FCH‐C3‐A** material. The two sublattices are characterized by different coercive fields and accordingly show different temperature and frequency dependencies. Similar measurements on a molecular derivative **FCH‐E** with only a single dipolar moiety further confirmed the ferroelectric properties of this family of materials and allowed an assignment of the coercive fields to the specific dipolar moieties. Integrating up the apparent switching currents obtained in the DWM measurements led to abnormally high and non‐saturating “polarization” hysteresis loops, with “remnant polarization” values far exceeding the dipolar density of the material. This behavior was explained in terms of a modulation of the conductivity by the ferroelectric polarization. As it was previously shown that charge transport in **FCH‐C3‐A**, which lacks an extended π‐system, differs from that in conventional organic semiconductors, this finding generalizes the polarization‐modulation of the electrical conductivity, which was previously reported in conventional organic semiconducting ferroelectrics, to this new class of conducting materials. In doing so, this work supports the idea that the symmetry argument underlying polarization‐direction‐driven conductivity modulation is universal: at finite fields, currents running parallel and anti‐parallel to the polarization direction are not equivalent, giving, at least in systems with localized charges, rise to measurable effects that may be used in, for instance, novel memory devices.

Future work will, amongst others, focus on lowering the temperatures that are necessary to enable switching. A promising way forward is by molecular design and particularly by changing, most likely shortening, the aliphatic chains on the phenyl ring [38].

## Conflicts of Interest

The authors declare no conflicts of interest.

## Supporting information




**Supporting File 1**: advs74335‐sup‐0001‐SuppMat.pdf

## Data Availability

The data that support the findings of this study are available from the corresponding author upon reasonable request.
