# Supplementary material for: A Supramolecular Ferroelectric With Two Sublattices and Polarization Dependent Conductivity
Source: Adv Sci (Weinh). 2026 Feb 11;13(22):e10258. doi: 10.1002/advs.202510258 (PMC13088293; doi:10.1002/advs.202510258)
Supplement: Supplementary file 1 — Supporting File 1: advs74335‐sup‐0001‐SuppMat.pdf [file ADVS-13-e10258-s001.pdf]

Supplementary Information to

A supramolecular ferroelectric with two sublattices and polarization dependent conductivity

H. Mager[1], M. Litterst[1], Sophia Klubertz[1], Shyamkumar V. Haridas[2], Oleksandr Shyshov[2], M. von Delius[*,2] and M. Kemerink[*,1]

[1] Institute for Molecular Systems Engineering and Advanced Materials, Heidelberg University, Im Neuenheimer Feld 225, 69120 Heidelberg, Germany

[2] Institute of Organic Chemistry, University of Ulm, Albert-Einstein-Allee 11, 89081 Ulm, Germany

*Corresponding author e-mail: max.vondelius@uni-ulm.de; martijn.kemerink@uni-heidelberg.de

Contents

1 – Materials 2

2 – Sample fabrication and methods.. 2

3 – Additional measurements and analysis 4

Supplementary references 22

1 – Materials

Full compound names and structures:

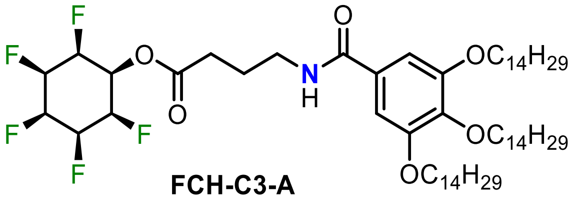

FCH-C3-A

FCH-C3-A is (1r,2R,3R,4s,5S,6S)-2,3,4,5,6-pentafluorocyclohexyl 3-(3,4,5-tris(tetradecyloxy)benzamido)butanoate

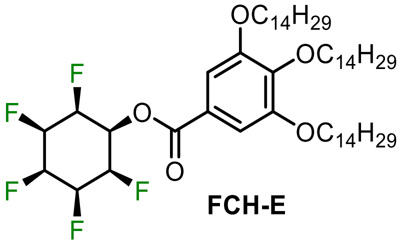

FCH-E

FCH-E is (1r,2R,3R,4s,5S,6S)-2,3,4,5,6-pentafluorocyclohexyl 3,4,5-tris(tetradecyloxy)benzoate

Materials were synthesized as described elsewhere.[1]

2 – Sample fabrication and methods

Substrate preparation and characterization: The thin film characterization was done on interdigitated electrodes (IDEs) which were either supplied by MicruX Technologies IDE or made by ourselves. For the former, the 180 pairs electrode pairs consist of 150 nm gold on top of 50 nm titanium deposited on a glass substrate. The total channel width, measured perpendicular to the direction of current flow, amounts to 0.8 m. The latter IDEs are produced by a UV photolithography process inside a cleanroom and consist of typically 25-30 nm gold on top of 3 nm chromium, both thermally evaporated on a glass substrate. Here, the total channel width amounts to 4 m. In both cases, the electrodes were 5 µm wide with a gap of 5 um between the electrodes, that is, the channel length is 5 µm.

Prior to active layer deposition, all substrates were cleaned the same way. The substrates were first mechanically washed with soap and water. Then, they were spaced out and put in succession into water, acetone and isopropanol for chemical cleaning. For each solution, the substrates were cleaned for 10 min at room temperature using an ultrasonic bath. Finally, the substrates were blown dry with nitrogen.

Film deposition: For reasons of material conservation, all samples were drop-casted from solution. **FCH-C3-A** and **FCH-E** were heated over the melting point and cooled back down before any measurement was taken. This was done to improve film homogeneity. **FCH-C3-A** and **FCH-E** were dissolved in tetrahydrofuran (THF) with concentrations between 10 to 20 mg/mL and drop-casted on glass substrates patterned with IDEs. To ensure full electrode

coverage, 2-5 times 4-5 µL were drop-casted as needed. No additional temperature treatment was used during the deposition process.

All samples had full electrode coverage with inhomogeneous films with film thicknesses typically ranging from 1 to 3 µm.

The capacitance-voltage (CV) and double wave method (DWM) measurements were taken using an aixACCT Systems Research Line DBLI together with a Linkam HFS600E-PB4 probe stage. For the CV measurements, a DC-bias is swept back and forth with a superimposed small AC-bias to measure the capacitance.

Surface characterization: Atomic force microscopy (AFM) images were obtained using a Bruker MultiMode 8-HR AFM operated in tapping mode.

Kelvin probe force microscopy: KPFM images were taken with an Oxford Instruments Jupiter XR Asylum Research AFM in sideband mode together with a Linkam HFS600E-PB4 probe stage for temperature control and device contacting. Voltages were applied by a Keithley 2636B System Source Meter.

Piezoresponse Force Microscopy: PFM images were taken with an Oxford Instruments Jupiter XR Asylum Research AFM operated in dual-AC resonance tracking (DART) mode.[2]

Structural analysis: The XRD measurements were carried out using a Rigaku SmartLab setup (Cu *source* with a 0.154 nm wavelength). The GIWAXS measurements were done in 2D mode with an incident angle of 0.30° and an exposure time of 3600 s.

Differential Scanning Calorimetry: DSC was conducted on Mettler Toledo DSC 2 STARe system under nitrogen atmosphere (heating and cooling was carried out at a rate of 5 °C/min).

3 – Additional measurements and analysis

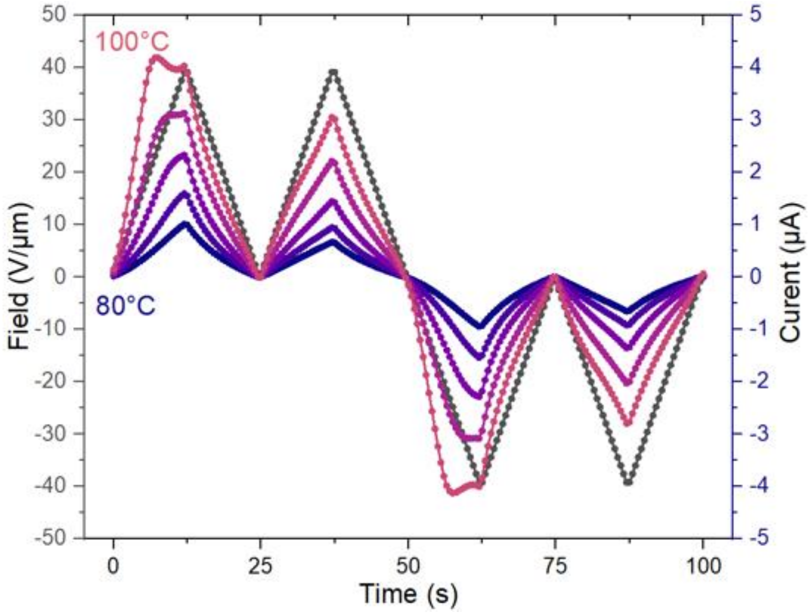

Figure S1: Zoom-in on lower temperatures in Fig. 2a, showing the emergence of the ferroelectric switching peak of **FCH-C3-A** at 90°C.

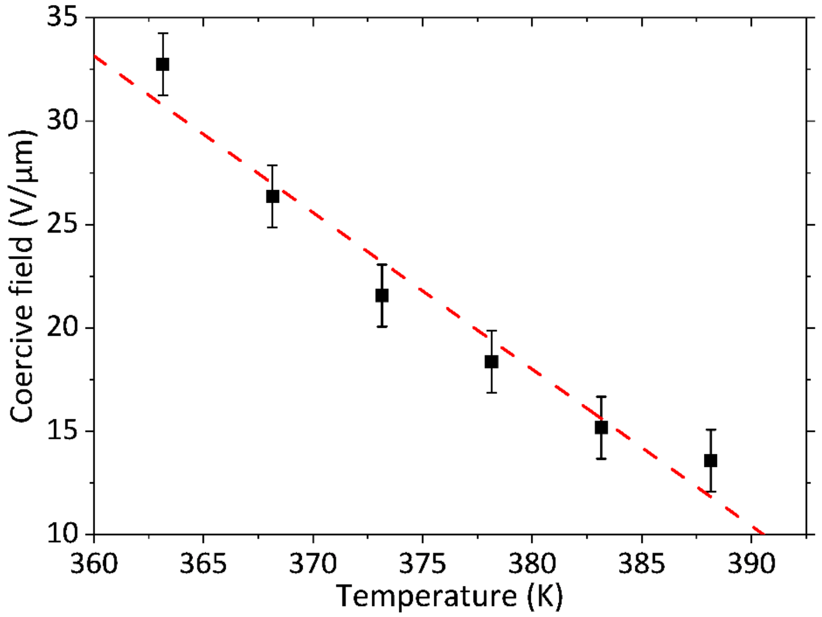

Figure S2: Coercive field values of **FCH-C3-A** (at 40 V/µm and 10 mHz) obtained from the field at the peak current in Fig. 2a plotted against temperature. The data is fitted with the TA-NLS model as described below.

Building on the Kalmogorov-Avrami-Ishibashi (KAI) model[3], Vopsaroiu *et al.* developed a model for polarization switching in ferroelectrics, based on thermally activated, nucleation limited switching (TA-NLS)[4,5]. For the coercive field, it gives the following dependence:

$$E_c = \frac{w_\mathrm{b}}{P_\mathrm{r}} - \frac{k_b T \cdot \ln(\nu_0 \tau \cdot \ln(2)^{-1})}{P_\mathrm{r} V^*} \qquad 1$$

Here w_b is the activation energy density of the critical nucleus required for polarization switching and V^* its volume. ν_0 is an attempt frequency, typical on the order of phonon frequencies of the material, P_r the remnant polarization and τ the rise time of the switching pulse. w_b and V^* are taken as fitting parameters, with an attempt frequency of 10 THz that is on the order of typical vibration frequencies in such materials. As explained later in the main text, the high field peak in the DWM measurements is assigned to the pentafluorocyclohexane group, resulting in a theoretical P_r value of 52 mC/m^2 and τ corresponds to 12 s for the measurement. This results in a w_b of 99 ± 1 meV/nm^3 and a V^* of 11 $\pm$ 1 nm^3. Both values are reasonable and are of similar magnitude as for the liquid crystalline BTA or the polymer P(VDF-TrFE).[6,7] It has to be noted, though, that w_b and V^* are in reality often better represented by distributions instead of by fixed parameters.[7] In addition, domain growth as described by the KAI model can be combined with the TA-NLS model for a more complete description. While incorporating the parameter distributions and the KAI model might correct for minor deviations from the TA-NLS model seen in our data, it is beyond the scope of this article.

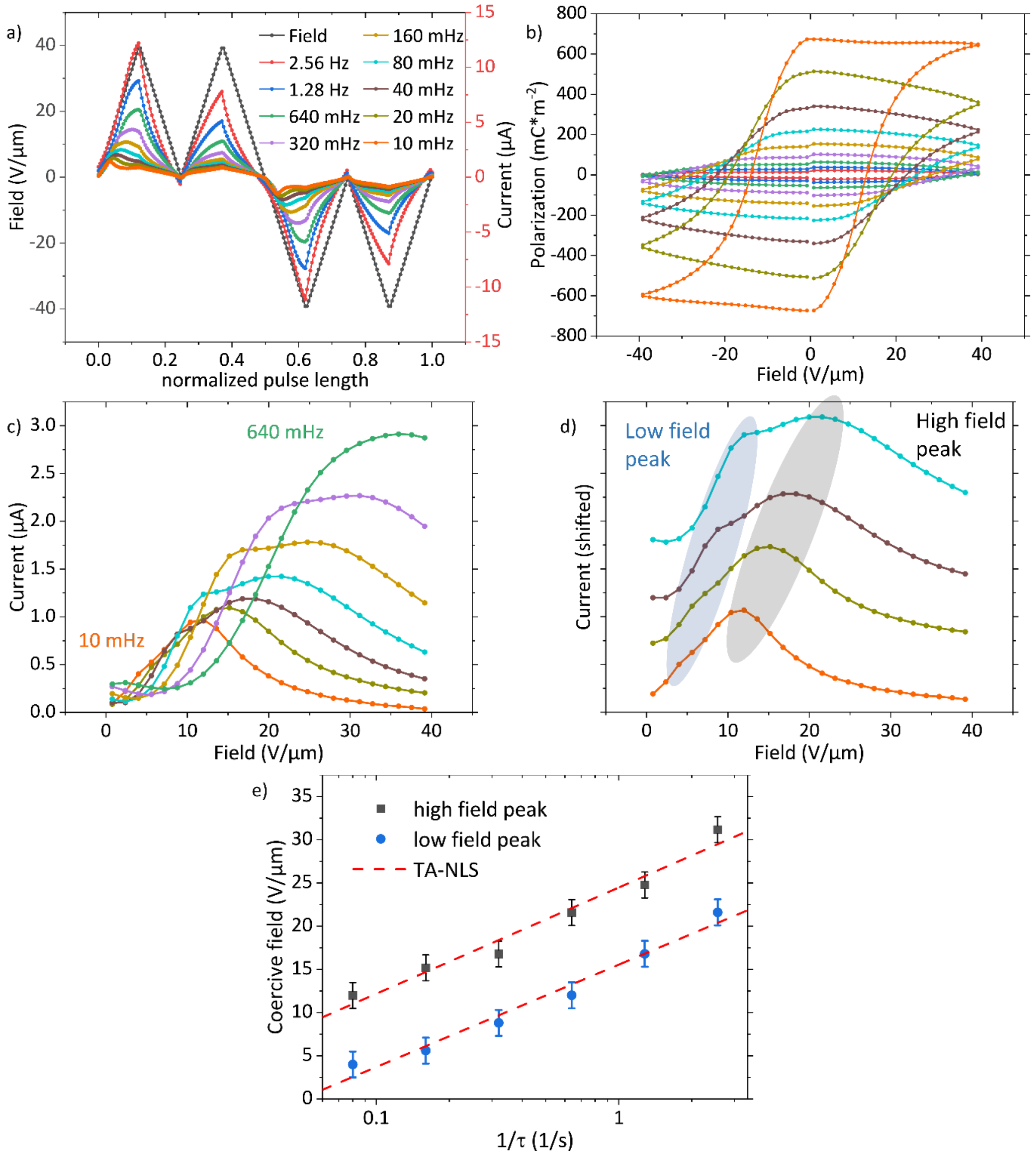

Figure S3: a) Frequency-dependent DWM measurements of **FCH-C3-A**, measured at 110°C. The x-axis is normalized to the pulse length. The lowest measurement frequency is 10 mHz which is then doubled for every measurement. Above 80 mHz parts of the switching peak are cut off. b) shows the corresponding hysteresis loops. c) shows the current transients obtained after DWM correction of a) and d) is a zoom-in to lower fields where two separate peaks are clearly visible. e) depicts the two coercive fields obtained from c), plotted against the inverse rise time, fitted with the TA-NLS model (equation 1). The fixed fitting parameters are an attempt frequency of 10 THz, a temperature of 383.15 K and the theoretical polarization values of 31 mC/m^2 and 52 mC/m^2, for the low and high field peak, respectively. Comparisons to the coercive field behavior of FCH-E and with the TA-NLS fitting parameters obtained in Fig. S11 indicate that the low field peak belongs to the amide group and the high field peak to the pentafluorocyclohexane group. The obtained fitting parameters for the low field peak are an energy barrier w_b of 33.1 $\pm$ 0.3 meV/nm^3 and a nucleation volume V^* of 33 $\pm$ 3 nm^3. For the

high field peak w_{b} of 60.4 ± 0.5 meV/nm^3 and a nucleation volume V^* of 19 ± 2 nm^3 are obtained. The high-field peak values are reasonably consistent with the values found for the temperature dependence in Fig. S2.

Since the peaks in the DWM are conductivity peaks and not switching peaks, the lack of a visible low field peak at lower temperatures in the DWM (cf. Fig. 2a) is explained by the presence of a finite injection barrier, which is only overcome once the pentafluorocyclohexane group becomes mobile as well. This is consistent with the injection barrier limited behavior seen at low fields in Fig. 5b. If this case, the amide switching peak should be observed instead. The reason the amide switching peak is not detected can be explained by the fact that compared to the general magnitude of the measured current, the amide switching current portion is small and therefore drowned out (cf. Fig. S1**)**.

In contrast, the CV measurements depicted in Fig. 3 show actual reversible ferroelectric switching, therefore the low field peak of the amide group is already visible at lower temperatures, independent of injection barriers. Reversible switching of the pentafluorocyclohexane group is assumed to be energetically costlier, meaning higher temperatures/fields and lower frequencies are required.

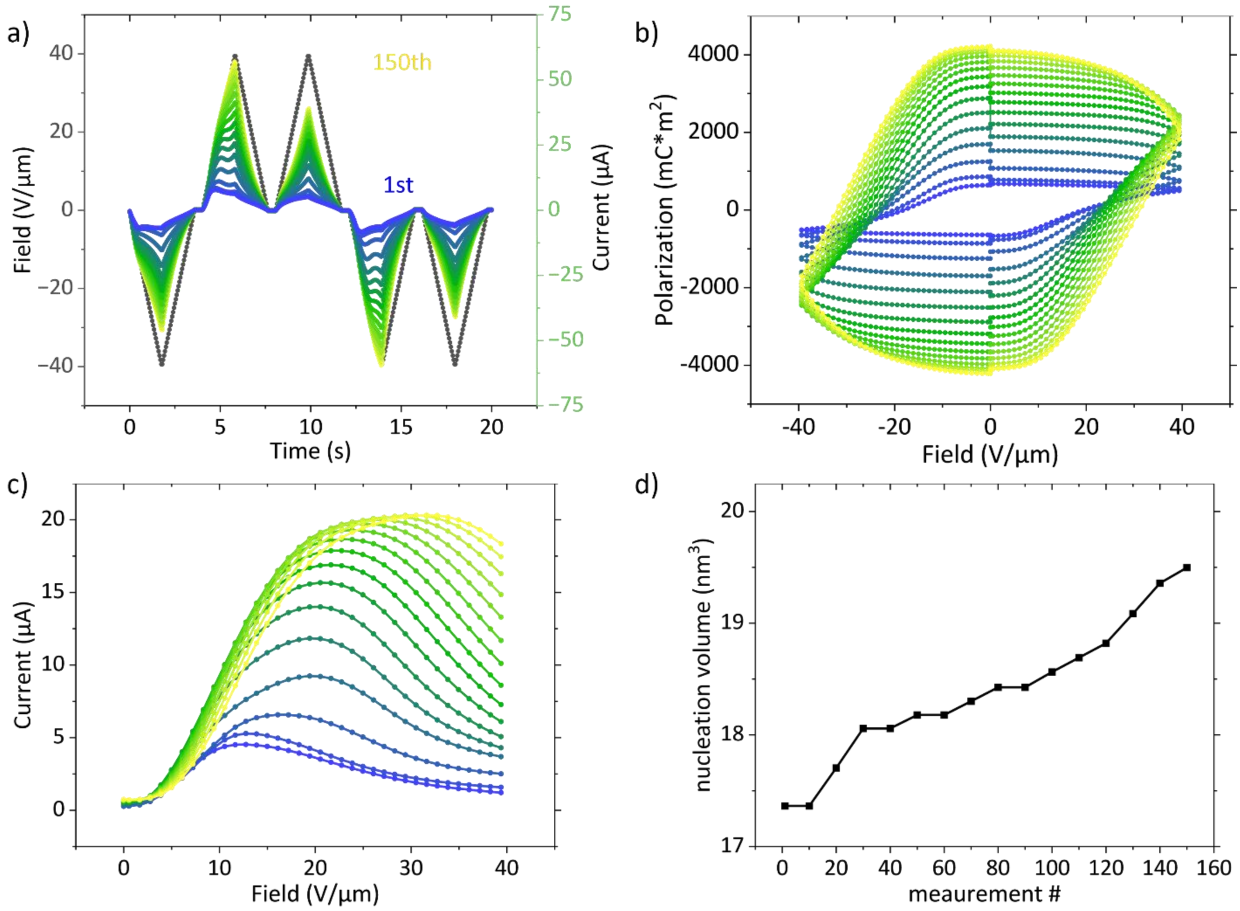

Figure S4: a) 150 consecutive DWM measurements of **FCH-C3-A** at fixed parameters of 60 mHz, 100°C and 40 V/µm. Only every tenth measurement is plotted. b) Corresponding "polarization" hysteresis loops, which keep growing as a result of the increasing conductivity. d) Obtained current transients after DWM background correction. c) Critical nucleation volume calculated for the coercive fields obtained and an energy barrier w_b of 60 meV/nm^3 obtained via the TA-NLS model.

Fig. S4 shows the effect of 150 consecutive measurements at fixed field, temperature and frequency on the switching and conducting properties. Two significant changes in the current response can be observed. On the one hand, the switching peak shifts to higher electric fields/later timestamps while on the other hand the background current increases by approximately one order of magnitude. As a result, part of the (apparent) switching current is cut off in later measurements, a higher field or lower frequency is required for full polarization switching at this point. The concomitant strong increase of the background current starts drowning out what remains of the switching peak. Irrespective of the (polarization-dependent) conductivity mechanism, the explanation for these trends can be traced to the morphological change of the material during measurements. In previous work, the **FCH-C3-A** was shown to form supramolecular fibers during field annealing (see Fig. 1e), which was accompanied by a significant rise in conductivity, followed by saturation and a soft roll-off.[1] While the fiber formation enhances material conductivity and therefore the background currents in DWM measurements, the shift of the switching peak towards higher fields additionally suggests that at the same time the dipole switching is progressively made more

difficult. This can be readily explained in terms of the TA-NLS model, where with increasing order and supramolecular structure, the critical nucleation volume increases, which in turn requires progressively larger electric fields to overcome the potential barrier between ferroelectric states. The evolution of the critical nucleation volume obtained from the TA-NLS is shown in Fig. S4d. In short, as its structural order increases, the material transitions to slightly more intrinsic (but still extrinsic) switching behavior. Melting the material reverts the structural changes and resets the conducting and ferroic properties. While the change in ferroelectric and conducting properties allows for a certain tunability, it also makes an unambiguous determination of the ferroelectric characteristic parameters difficult, as they are heavily dependent on the sample's current morphology and therefore measurement history. Fully aligning to obtain a comparable base is not an option, as the resulting conductivity overshadows the ferroelectric effects. For comparability's sake, all data shown is collected from more or less pristine samples.

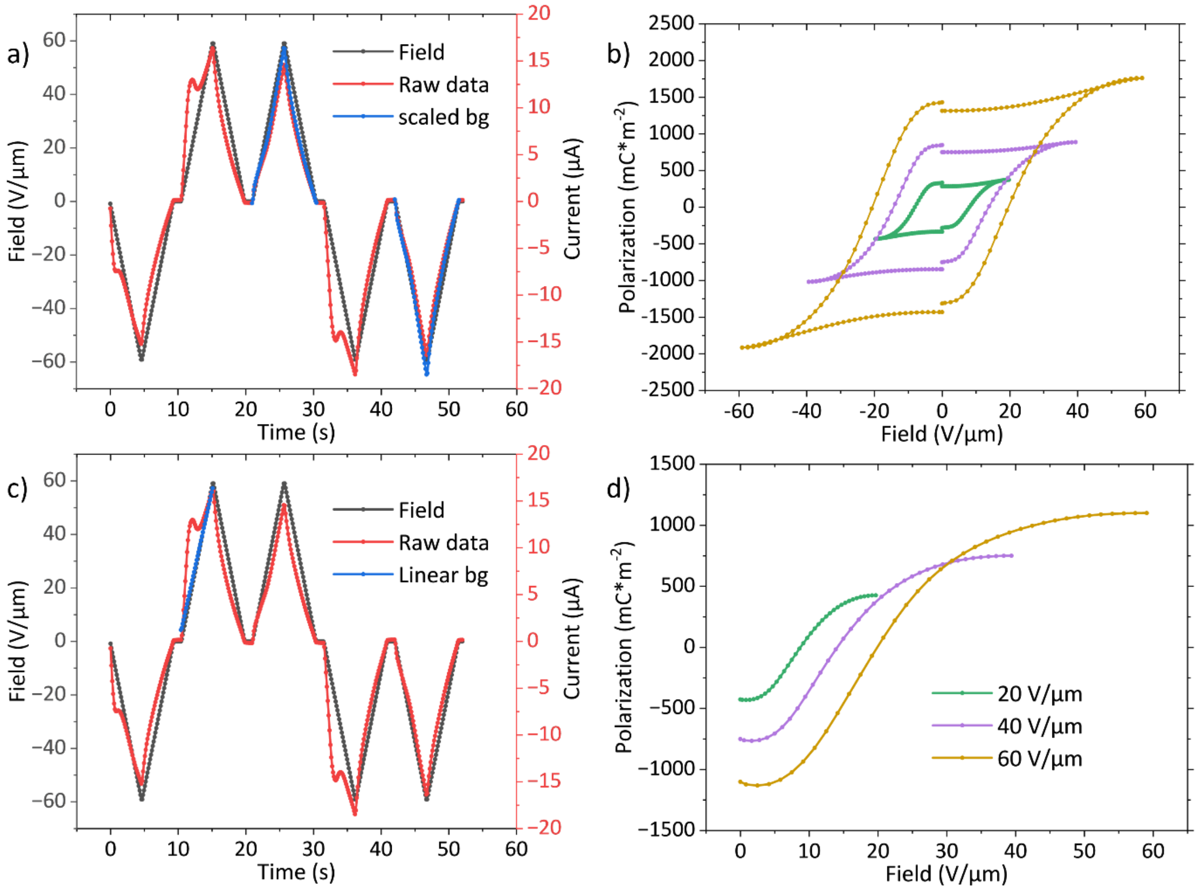

Figure S5: Different ways of background current correction are shown in an attempt to extract the contribution by true polarization reversal from the total apparent switching current. In a) a linear background current is subtracted from the current of the rising flank with the resulting integrated charge plotted on the right. As the resulting charge still vastly exceeds to theoretically expected polarization charge, this background correction is insufficient. Going one step further, the non-switching peaks in b) are scaled to the same value as the background current peak in the switching peaks. This should account for transient current effects. Subtraction of the scaled peaks from the switching peaks and integrating gives the "polarization" plotted on the right. Although reduced, its magnitude is still too large to be the result of dipolar switching.

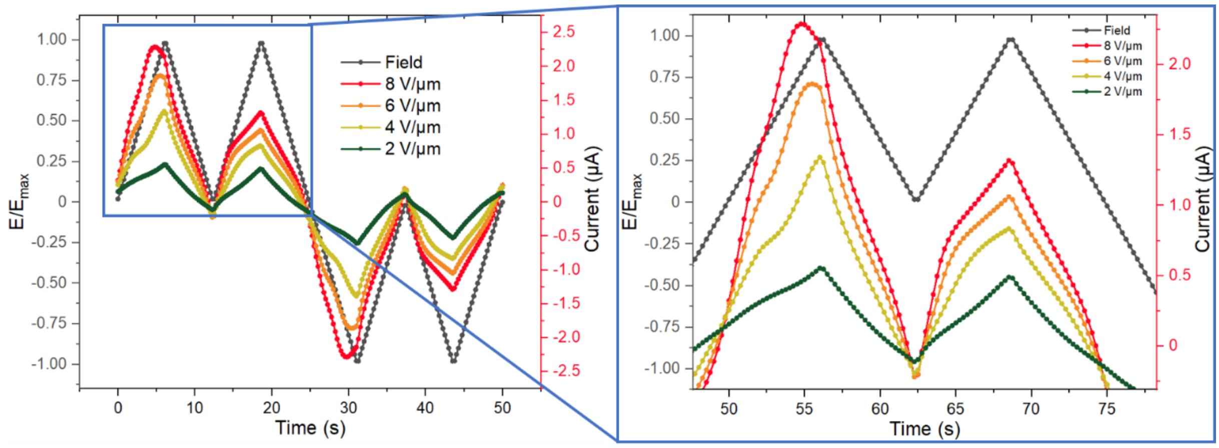

Figure S6: Low field DWM on **FCH-C3-A** at 110°C and 20 mHz. In the zoom-in on the right two shoulders can be clearly seen in the first pulse of the 6 V/µm line. We attribute the presence of two shoulders to the partially separate switching of the two dipolar moieties of the molecule, that is also observed in the CV measurements in Fig. 3 and in the frequency dependent DWM in Fig. S3. The shoulder in the second peak is attributed to incomplete polarization switching in the first peak.

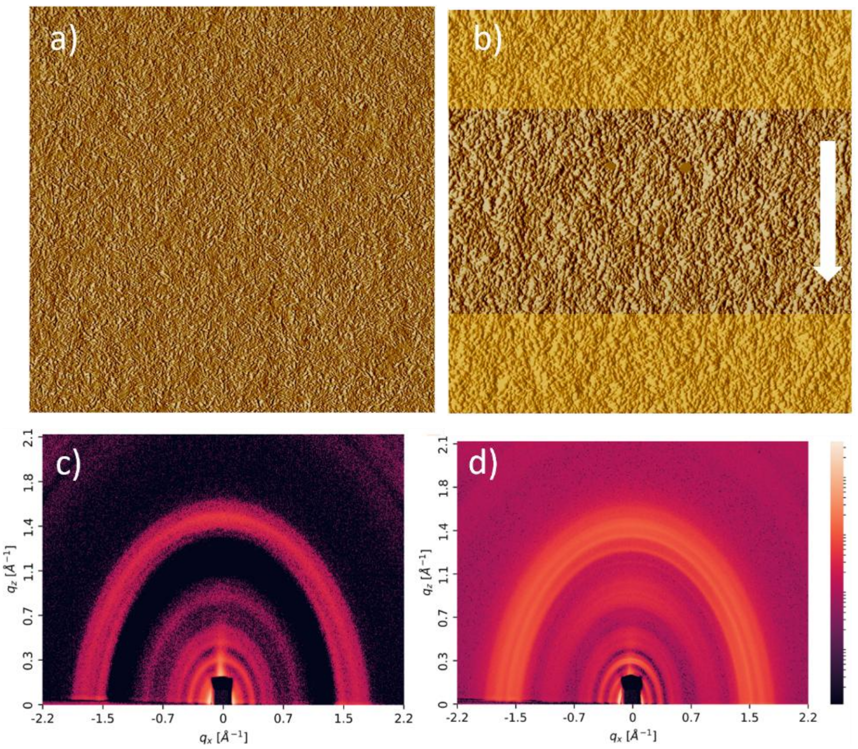

Figure S7: a) and b) show atomic force microscopy amplitude channel images of a **FCH-E** thin film before and after annealing at 90°C for one hour with an applied field of 20 V/µm. The buried electrode structure is indicated by the golden bars and the white arrow shows the field direction. c) and d) show corresponding grazing incident wide angle scattering images.

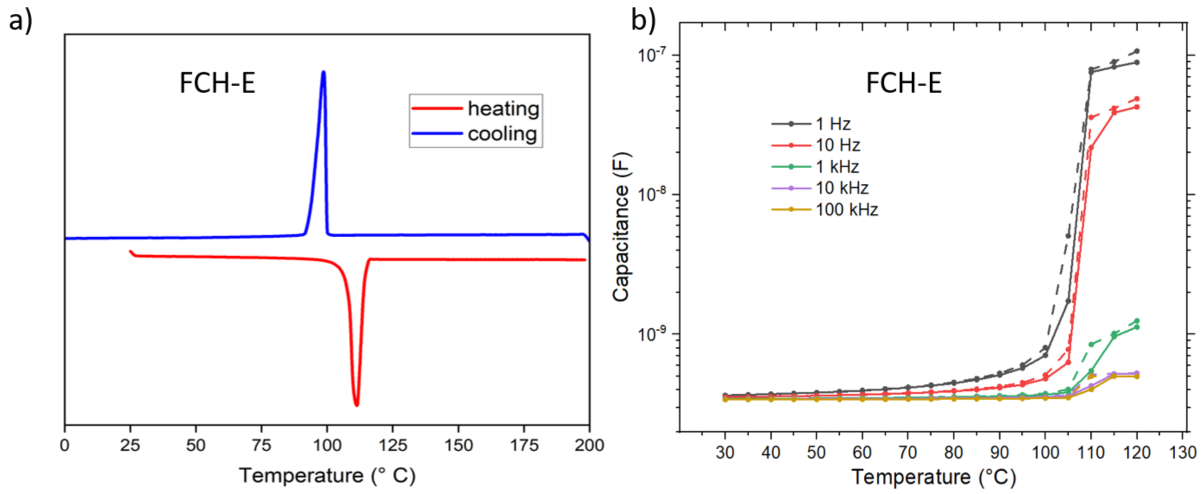

Figure S8: Phase behavior of **FCH-E**. a) DSC traces measured under nitrogen atmosphere. Only one peak at around 110°C in the heating trace associated with the melting of the material is observed. The corresponding peak of the freezing point is at 95°C. b) Dielectric spectroscopy measurements, exhibiting a stepwise capacitance increase at the melting point and no other features.

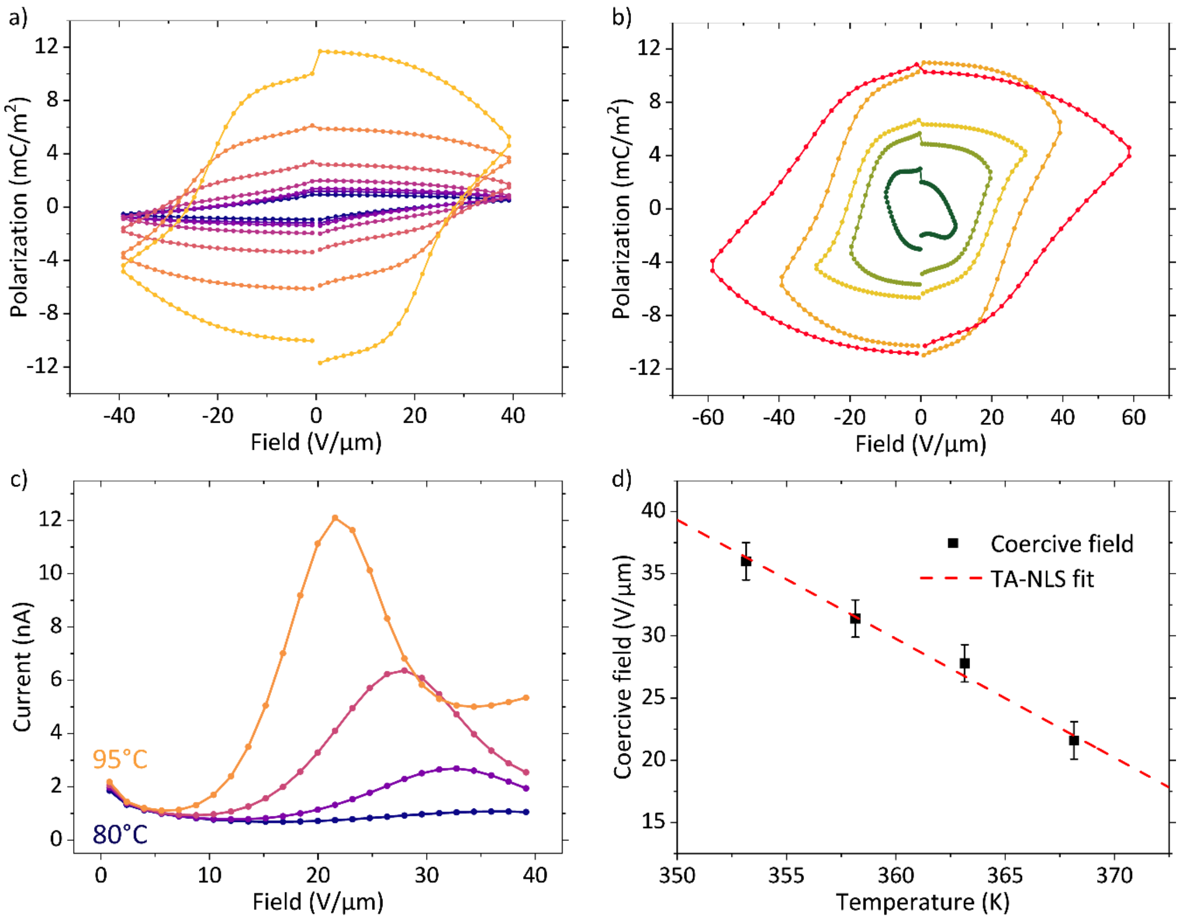

Figure S9: a) and b) are the corresponding polarization hysteresis loops to the DWM measurements on **FCH-E** at 20 mHz as shown in Fig. 4a) and b). Although far from ideal, both panels show indications for polarization saturation for increasing electric fields. However, compared to a theoretical polarization of 69 mC/m^2 for a fully polarized fluorinated cyclohexane ring, the observed polarization values around 5 mC/m^2 indicate that only a partial reorientation of the fluorine rings occurs. c) shows the current transients obtained from the temperature dependent measurements in Fig. 4a) after DWM correction. The corresponding coercive fields are plotted against the temperature in d) and fitted with the TA-NLS model, taking a typical attempt frequency of 10 THz, the theoretical polarization value of 69 mC/m^2, and a rise time of $\tau = 6$ s. The resulting fitting parameters are the nucleation volume $\mathrm{V}^* = 6.7 \pm 0.3$ nm^3 and $w_b = 161 \pm 6$ meV, which are in line with other organic ferroelectrics.[6] Comparing the coercive field values with those at the corresponding temperature for the **FCH-C3-A** material, that is, ~20 K higher due to the higher melting point, reinvigorates the attribution of the high-field peak in Fig. S3 to the pentafluorocyclohexane group.

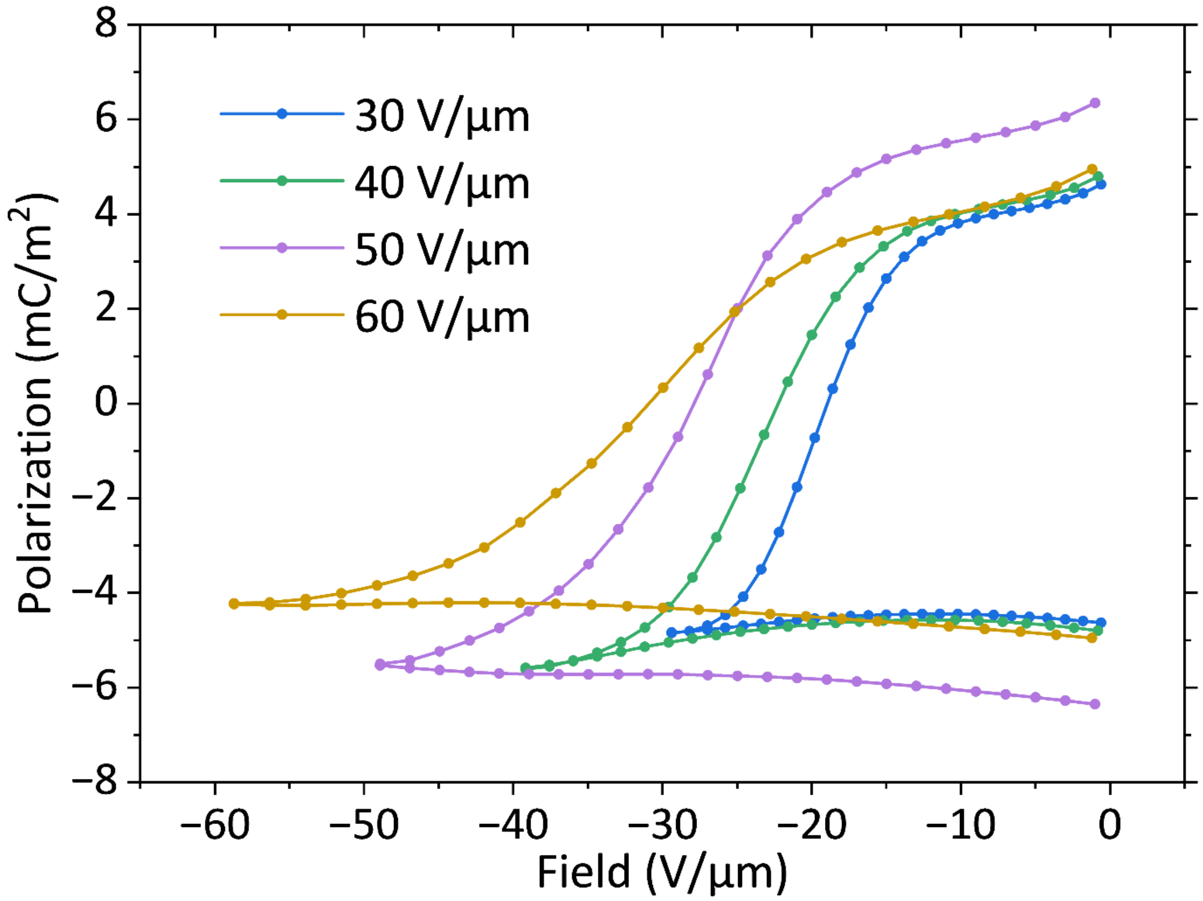

Figure S10: Negative part of the polarization hysteresis loop obtained via DWM of **FCH-E** from Fig. 4b) after scaling the background current as schematically shown in Fig. S5a). The resulting polarization is assumed to be the actually measured ferroelectric polarization and amounts to 5.1 ± 0.8 mC/m^2. For comparison, the approximate theoretical upper limit for a fully flipped all-cis fluorinated cyclohexane group is ~69 mC/m^2.

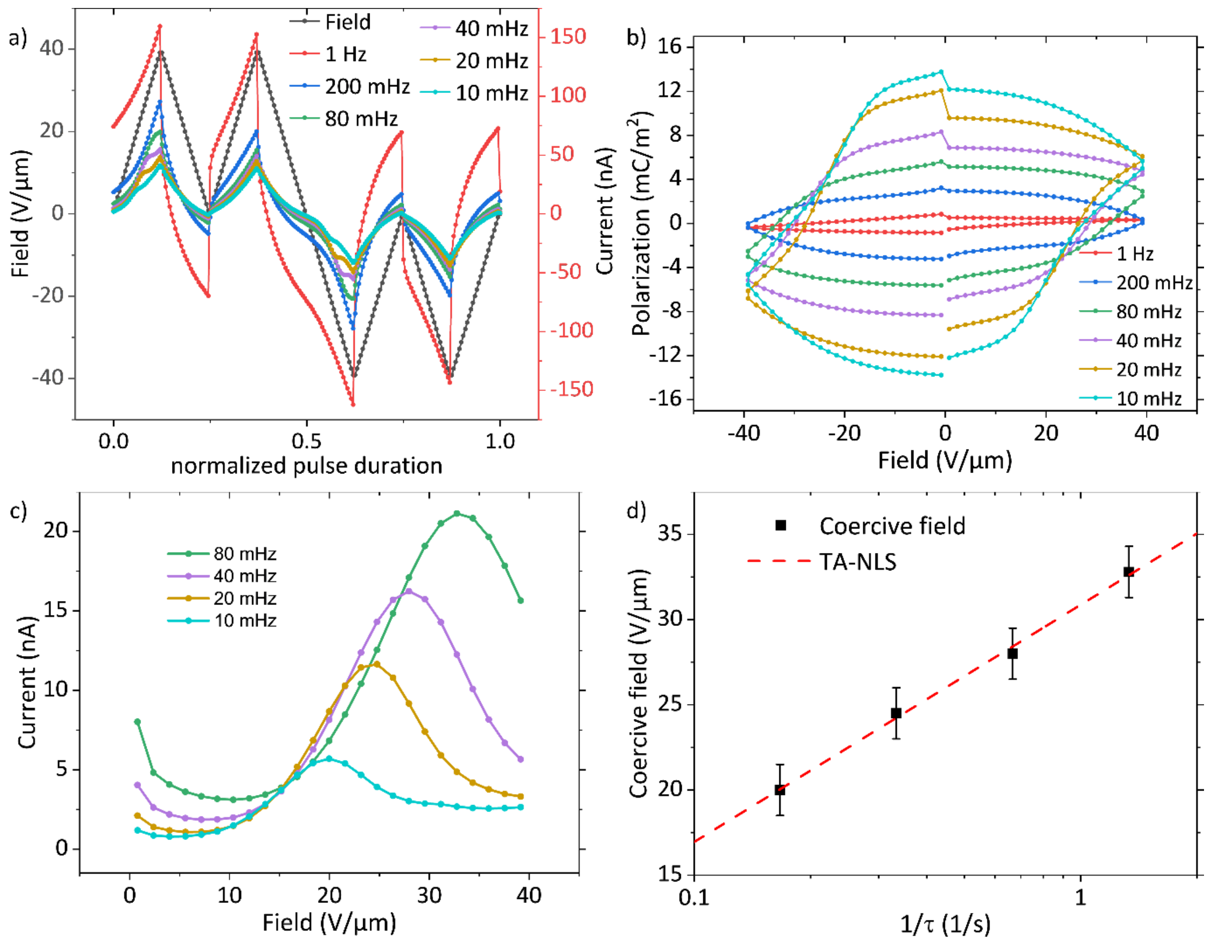

Figure S11: a) shows DWM measurements of **FCH-E** at varying frequency at fixed field and a temperature of 95°C with the corresponding hysteresis loops depicted in b). c) shows the current transients obtained after the DWM background correction. The corresponding coercive fields are plotted against the frequency and fit with the TA-NLS model in d). The obtained fitting parameters are an energy barrier w_b of 99.2 ± 0.3 meV/nm^3 and a nucleation volume V^* of 12 ± 2 nm^3. These values agree well with those obtained in Fig. S3 for the high field peak of FCH-C3-A, attributed to the fluorinated cyclohexane group. This further reinforces our peak assignment to each dipolar moiety.

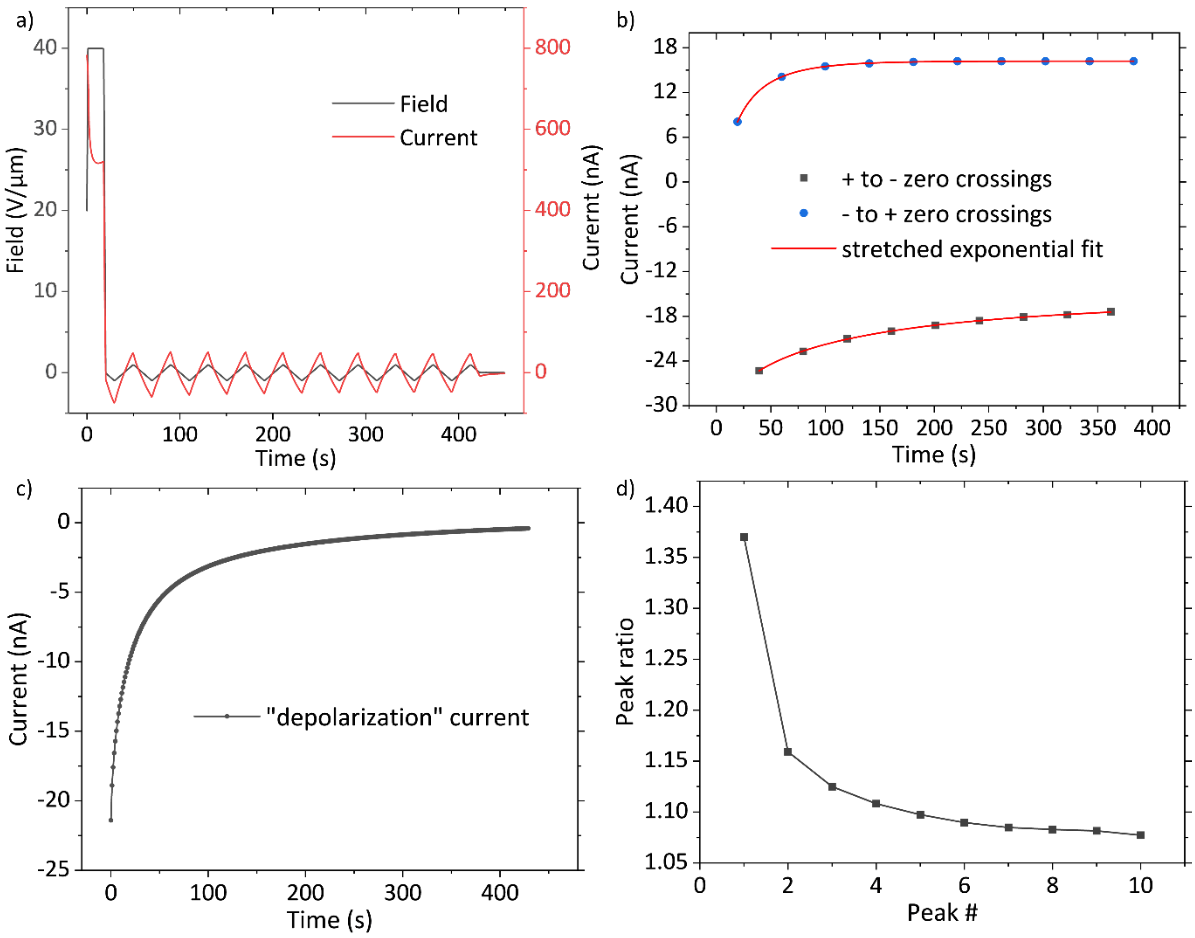

Figure S12: Illustration of the steps taken to extract the peak ratios plotted in Fig. 5b) from conductivity measurements on **FCH-C3-A** as depicted in a). First, the currents at the zero crossings of the voltage are obtained and plotted exemplary in b), from positive to negative and vice versa. These are composed of the depolarization current in response to the large rectangular poling pulse, further transient currents like ionic contributions and the displacement current resulting from the field sweep rate. The currents are fitted with a stretched exponential, which is commonly used to describe depolarization processes[8–10], while an added constant term describes the displacement current. With the fit parameters, the depolarization current is reconstructed to the starting time $t = 0$ by averaging the two traces obtained from crossing zero voltage in either direction. This additionally gets rid of the displacement current. An example of a resulting "depolarization" current is plotted in c). The charge obtained by integrating the current transients obtained from multiple measurements averages to around 270 mC/m^2 and varies between 125 mC/m^2 and 356 mC/m^2. As the obtained charge is of the same order but larger than what can solely originate from depolarization (cf. theoretical dipole density of 83 mC/m^2), ionic currents or other transients or offset errors are apparently not corrected for. It has to be noted that, as the functional shape is a stretched exponential decay, small variations in starting time result in large differences of total integrated charge, as do any offset errors. Finally, a) is corrected by the transient current background shown in c), and the peak conductivity ratios of the nth peak, that are modulated by the ferroelectric polarization, are shown in d).

Discussion of errors in current modulation. Given the modest values of the ferroelectric-driven conductivity modulation in Figs. 5b, S12d and S13d (below), it is important to assess the corresponding error bars. In general, the subtracted background currents are significantly smaller than the conduction current of interest, which can be seen by comparing the currents at zero voltage in Fig. S12b (<20 nA) to the peak currents in Fig. S12a (>50 nA). After a few tens of seconds, the background currents consist mostly of (anticipated) displacive contributions (plateau values in Fig. S12b, around ±18 nA) that can be removed by averaging up- and down-going zero crossings, as discussed above. As illustrated in SI Fig. S12c, the remaining 'spurious' (depolarization) current drops quickly to a few nA. Hence, the fact that we find a finite and significant conductivity modulation does not depend critically on the procedure for correction, even if its absolute value does. We note that the uncorrected data would give a larger modulation than shown in Figs. 5b and S12d, due to the depolarization current being negative.

To estimate an (overly generous) upper limit for the resulting error bars, one could use the ratio of the depolarization current and the total peak current at maximum reading voltage. This ratio would be larger for early peak pairs and smaller reading voltages and significantly drop off for later peak pairs and higher reading voltages. As an example, the peak current for the smallest applied voltage of 1 V, i. e. a field of 0.2 V/µm, are around 5 nA, giving an error bar around 100% for the first peak pair. However, for later pairs, it drops to below 10%. The fact that the modulation ratio (Fig. 5b) is basically constant for all peak pairs illustrates that these error estimates are likely an overestimation. For larger reading voltages, e.g. 5 V (1 V/µm), peak currents are much larger (~50 nA) and correspondingly error bars are much smaller (5-10% for the first peak pair, dropping to a few % or less for later pairs).

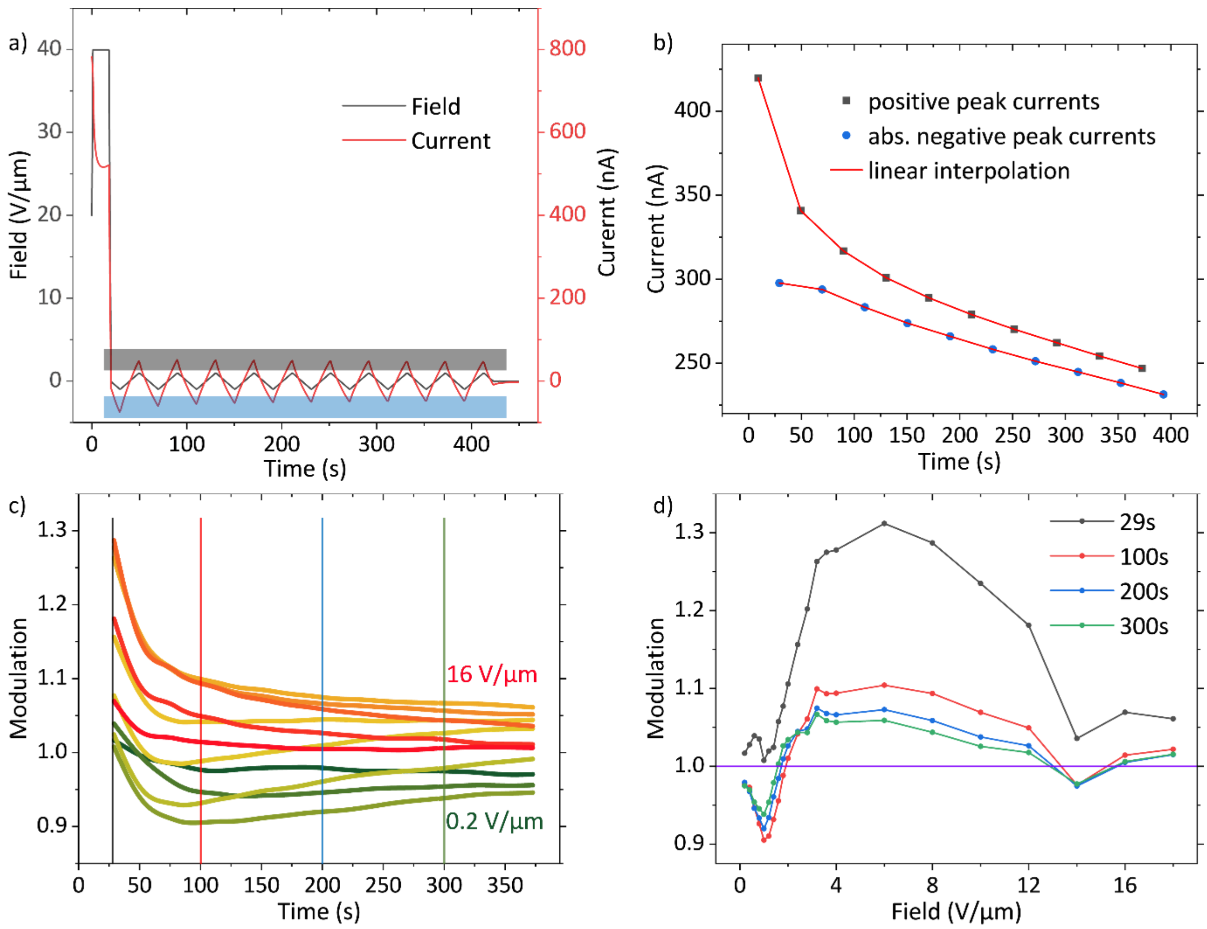

Figure S13: Alternative approach to obtain the conductivity modulation in **FCH-C3-A** without the time shift of the up and down peaks. Again, the transient background currents and the displacement current are extracted from the zero crossings of a) as depicted in Fig. S12. The corrected current values of the peaks in a) are plotted in b) and linearly interpolated. From those the current modulation over time shown in c) is obtained for all fields. The modulations were smoothed to account for the linear interpolation edges. The vertical lines indicate the timestamps of 29s, 100s, 200s and 300s for which the field dependence of the modulation is depicted in d), which show an analog behavior as the peak ratios in Fig. 5b).

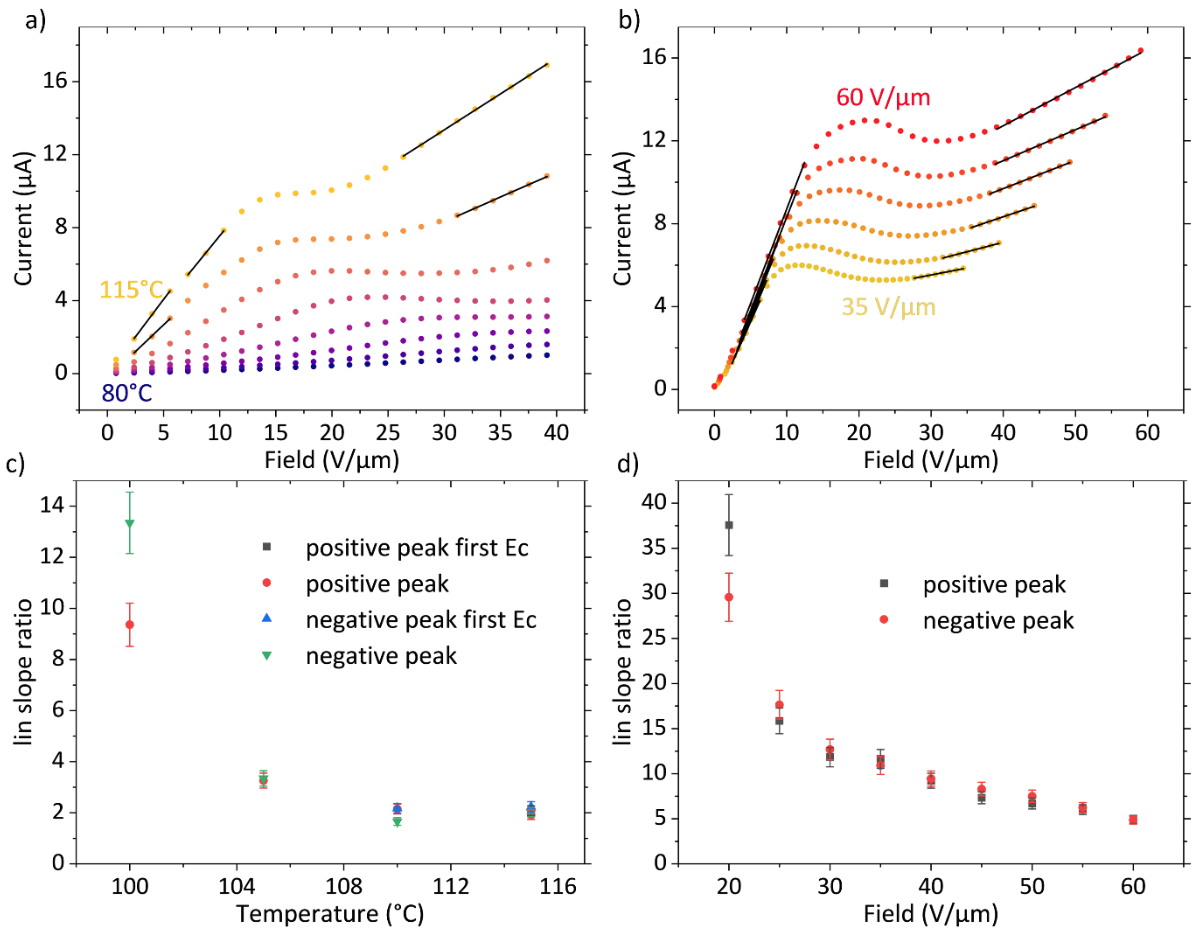

Figure S14: On-off ratios obtained from the DWM measurements on **FCH-C3-A** shown in Fig. 2a) and 2c). To that end the left flanks of the switching pulses, i.e. the first positive and first negative (i.e. third overall) pulse, where fitted linearly below and above the coercive field, as depicted in a) for the temperature dependent case (data from Fig. 2a) and in b) for the field dependent case (data from Fig. 2c). In the former, the two different coercive fields have to be kept in mind and are fitted separately, while in the data of the latter only the second, high field coercive field is visible. The ratios obtained by dividing the current slope below the coercive field by the current slope beyond the coercive field are shown in c) and d). Especially for lower fields and temperatures, the switching process might not be completely finalized, resulting in smaller slopes above the coercive field and an inflated on/off-ratio. Error margins are obtained by estimating the variation by changes of the fitting range.

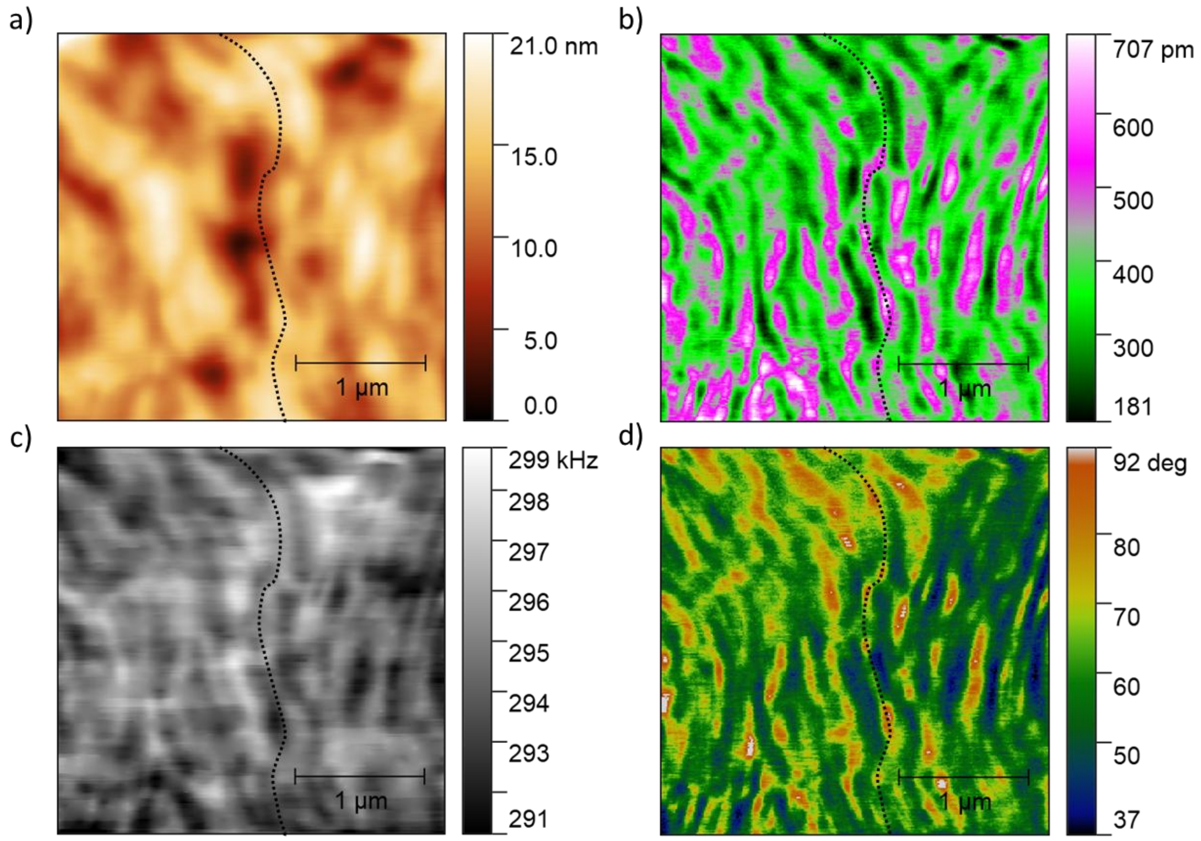

Figure S15: Dual-AC resonance tracking (DART) PFM measurements on a pristine FCH-C3-A film, spincoated on an ITO coated glass substrate. Measurements were carried out at room temperature under ambient conditions: a) topography, b) amplitude, c) contact frequency, d) phase. Note that the width of the fibrillar features is ~100 nm, hence these are likely to reflect a ternary structure consisting of bundles made up of multiple supramolecular polymers, which only have a width of a few nm each. The dotted line is the same in all panels and traces a single fibrillar feature. Detailed information about DART-PFM can be found in Ref. [2].

Discussion of PFM experiments. Although a popular technique to demonstrate ferroelectricity, PFM is not a straightforward technique, especially on soft (organic) materials.[11] A fundamental and additional problem in the current system is that the fiber bundles, and hence the preferential axis of polarization, lay in-plane after deposition while the probing field between tip and the (in this experiment needed) conductive substrate is cross-plane. Hence, to see any switching effect in PFM, one would first have to use the tip-field to re-orient the supramolecular fibers – and do so without just digging a hole, i.e., destroying the sample and contaminating the tip. Hence, we turned to a passive, i.e., non-switching PFM experiment in which we aim to probe (but not switch) any spontaneous polarization that might be there: since one needs a conductive substrate for the PFM experiment, it is not possible to field-align and/or pole the sample in the in-plane direction as done in the experiments on IDEs, so one has to rely on spontaneous polarization. The latter may be anticipated to consist of domains of alternating orientation (such that they average to zero) with a preferential axis along the fibrils. Importantly, even if the domains are sufficiently large to be resolved, this would only show the presence of finite piezoelectric constants, but not that any underlying macroscopic dipole can be field-switched. Because of the complicated geometry of twisted in-plane fibrils

and an inhomogeneous and largely cross-plane field, the signal would contain a mixture of on- and off-diagonal tensor elements (d_{33}, d_{13} etc.).

The results of this passive PFM analysis are shown in Fig. S15. Since there is no evident correlation between topography (panel a) and piezo (panels b-d) signals, it seems unlikely that the features in the latter are a mere crosstalk effect. Crucially, there is a binary phase contrast in the fibrillar structures (panel d) that does not one-on-one correlate with the amplitude (panel b) and/or the frequency (panel c), as can be seen by tracing individual fibrils, cf. the dotted lines. As discussed above, this phenomenology is what one would expect for domains with opposite polarization directions in a quasi-1D fibrillar morphology. Hence, we take the PFM results as supportive of the idea that the dipolar groups in **FCH-C3-A** combine into macroscopic dipoles with a well-defined axis of polarization, most likely along the fibril axis.

Supplementary references

(1) Mager, H.; Butkevich, A. A.; Klubertz, S.; Haridas, S. V.; Shyshov, O.; Wakchaure, V. C.; Borstelmann, J.; Michalsky, I.; García-Iglesias, M.; Rodríguez, V.; González-Rodríguez, D.; Palmans, A. R. A.; Kivala, M.; Delius, M. von; Kemerink, M. Long-Range Electrical Conductivity in Non-π-Conjugated Organic Molecular Materials. arXiv June 3, 2025. https://doi.org/10.48550/arXiv.2506.02673.

(2) Rodriguez, B. J.; Callahan, C.; Kalinin, S. V.; Proksch, R. Dual-Frequency Resonance-Tracking Atomic Force Microscopy. *Nanotechnology* **2007**, *18* (47), 475504. https://doi.org/10.1088/0957-4484/18/47/475504.

(3) Ishibashi, Y.; Takagi, Y. Note on Ferroelectric Domain Switching. *J. Phys. Soc. Jpn.* **1971**, *31* (2), 506–510. https://doi.org/10.1143/JPSJ.31.506.

(4) Vopsaroiu, M.; Blackburn, J.; Cain, M. G.; Weaver, P. M. Thermally Activated Switching Kinetics in Second-Order Phase Transition Ferroelectrics. *Phys. Rev. B* **2010**, *82* (2), 024109. https://doi.org/10.1103/PhysRevB.82.024109.

(5) Vopsaroiu, M.; Weaver, P. M.; Cain, M. G.; Reece, M. J.; Kok Boon Chong. Polarization Dynamics and Non-Equilibrium Switching Processes in Ferroelectrics. *IEEE Trans. Ultrason., Ferroelect., Freq. Contr.* **2011**, *58* (9), 1867–1873. https://doi.org/10.1109/TUFFC.2011.2025.

(6) Urbanaviciute, I.; Bhattacharjee, S.; Biler, M.; Lugger, J. A. M.; Cornelissen, T. D.; Norman, P.; Linares, M.; Sijbesma, R. P.; Kemerink, M. Suppressing Depolarization by Tail Substitution in an Organic Supramolecular Ferroelectric. *Phys. Chem. Chem. Phys.* **2019**, *21* (4), 2069–2079. https://doi.org/10.1039/C8CP06315J.

(7) Urbanavičiūtė, I.; Cornelissen, T. D.; Meng, X.; Sijbesma, R. P.; Kemerink, M. Physical Reality of the Preisach Model for Organic Ferroelectrics. *Nat Commun* **2018**, *9* (1), 4409. https://doi.org/10.1038/s41467-018-06717-w.

(8) Jo, W.; Kim, D. C.; Hong, J. W. Reverse-Poling Effects on Charge Retention in Pb(Zr,Ti)O3(001)/LaNiO3(001) Heterostructures. *Appl. Phys. Lett.* **2000**, *76* (3), 390–392. https://doi.org/10.1063/1.125763.

(9) Hong, J. W.; Jo, W.; Kim, D. C.; Cho, S. M.; Nam, H. J.; Lee, H. M.; Bu, J. U. Nanoscale Investigation of Domain Retention in Preferentially Oriented PbZr0.53Ti0.47O3 Thin Films on Pt and on LaNiO3. *Appl. Phys. Lett.* **1999**, *75* (20), 3183–3185. https://doi.org/10.1063/1.125271.

(10) V. Gorbunov, A.; Meng, X.; Urbanaviciute, I.; Putzeys, T.; Wübbenhorst, M.; P. Sijbesma, R.; Kemerink, M. Polarization Loss in the Organic Ferroelectric Trialkylbenzene-1,3,5-Tricarboxamide (BTA). *Physical Chemistry Chemical Physics* **2017**, *19* (4), 3192–3200. https://doi.org/10.1039/C6CP08015D.

(11) Litterst, M.; Butkevich, A. A.; Kemerink, M. Inconclusive Proof of Ferroelectricity in Peptide-VDF Ribbons. *Nature* **2025**, *644* (8075), E1–E3. https://doi.org/10.1038/s41586-025-09314-2.
